# Diagnosis of paediatric tuberculosis by optically detecting two virulence factors on extracellular vesicles in blood samples

**DOI:** 10.1038/s41551-022-00922-1

**Published:** 2022-08-19

**Authors:** Wenshu Zheng, Sylvia M. LaCourse, Bofan Song, Dhiraj Kumar Singh, Mayank Khanna, Juan Olivo, Joshua Stern, Jaclyn N. Escudero, Carlos Vergara, Fangfang Zhang, Shaobai Li, Shu Wang, Lisa M. Cranmer, Zhen Huang, Christine M. Bojanowski, Duran Bao, Irene Njuguna, Yating Xiao, Dalton C. Wamalwa, Duc T. Nguyen, Li Yang, Elizabeth Maleche-Obimbo, Nhung Nguyen, Lili Zhang, Ha Phan, Jia Fan, Bo Ning, Chenzhong Li, Christopher J. Lyon, Edward A. Graviss, Grace John-Stewart, Charles D. Mitchell, Alistair J. Ramsay, Deepak Kaushal, Rongguang Liang, Eddy Pérez-Then, Tony Y. Hu

**Affiliations:** 1 Center for Cellular and Molecular Diagnostics, Tulane University School of Medicine, New Orleans, LA USA; 2 Department of Biochemistry and Molecular Biology, Tulane University School of Medicine, New Orleans, LA USA; 3 Department of Medicine, Division of Allergy and Infectious Diseases, University of Washington, Seattle, WA USA; 4 Department of Global Health, University of Washington, Seattle, WA USA; 5 James C. Wyant College of Optical Sciences, The University of Arizona, Tucson, AZ USA; 6 Southwest National Primate Research Center, Texas Biomedical Research Institute, San Antonio, TX USA; 7 Department of Microbiology, Immunology and Parasitology, LSU Health Sciences Center, New Orleans, LA USA; 8 O&M Medical School (O&Med), Santo Domingo, Dominican Republic; 9 Virginia G. Piper Biodesign Center for Personalized Diagnostics, The Biodesign Institute, Arizona State University, Tempe, AZ USA; 10 Department of Pediatrics, Division of Pediatric Infectious Diseases, Emory School of Medicine, Atlanta, GA USA; 11 Children’s Healthcare of Atlanta, Atlanta, GA USA; 12 Department of Epidemiology, Emory Rollins School of Public Health, Atlanta, GA USA; 13 Section of Pulmonary Diseases, Tulane University School of Medicine, New Orleans, LA USA; 14 Kenyatta National Hospital, Research and Programs, Nairobi, Kenya; 15 Department of Pediatrics and Child Health, University of Nairobi, Nairobi, Kenya; 16 Department of Pathology and Genomic Medicine, Houston Methodist, Houston, TX USA; 17 National Lung Hospital, Ha Noi, Vietnam; 18 Center for Promotion of Advancement of Society (CPAS), Ha Noi, Vietnam; 19 Vietnam National Tuberculosis Program/University of California San Francisco Research Collaboration, Ha Noi, Vietnam; 20 Department of Surgery, J.C. Walter, Jr. Transplant Center, Sherrie and Alan Conover Center for Liver Disease and Transplantation, Houston Methodist, Houston, TX USA; 21 Department of Pediatrics, University of Washington, Seattle, WA USA; 22 Department of Epidemiology, University of Washington, Seattle, WA USA; 23 Department of Pediatrics, Division of Infectious Diseases and Immunology, University of Miami Miller School of Medicine, Batchelor Children’s Research Institute, Miami, FL USA

**Keywords:** Tuberculosis, Diagnostic markers

## Abstract

Sensitive and specific blood-based assays for the detection of pulmonary and extrapulmonary tuberculosis would reduce mortality associated with missed diagnoses, particularly in children. Here we report a nanoparticle-enhanced immunoassay read by dark-field microscopy that detects two *Mycobacterium tuberculosis* virulence factors (the glycolipid lipoarabinomannan and its carrier protein) on the surface of circulating extracellular vesicles. In a cohort study of 147 hospitalized and severely immunosuppressed children living with HIV, the assay detected 58 of the 78 (74%) cases of paediatric tuberculosis, 48 of the 66 (73%) cases that were missed by microbiological assays, and 8 out of 10 (80%) cases undiagnosed during the study. It also distinguished tuberculosis from latent-tuberculosis infections in non-human primates. We adapted the assay to make it portable and operable by a smartphone. With further development, the assay may facilitate the detection of tuberculosis at the point of care, particularly in resource-limited settings.

## Abstract (web summary)

The optical detection of two virulence factors of *Mycobacterium tuberculosis* on extracellular vesicles in blood allowed for the diagnosis of tuberculosis in paediatric cases that were missed by clinical microbiological assays.

## Main

Tuberculosis (TB) is the second leading cause of death from infectious disease and a top-ten cause of death globally^1–3^, with an estimated 10 million new cases and 1.2 million deaths yearly. Despite sustained global efforts towards the control of TB, children aged less than 15 years account for ~1 million TB cases annually and for 230,000 TB-related deaths^4^. Most paediatric TB deaths (80%) occur in children less than 5 years old, and 96% of these deaths occur in children who are untreated owing to missed diagnoses^5,6^. The COVID-19 pandemic has hindered global TB-control efforts, and models from the World Health Organization (WHO) suggest that TB-notification decreases observed in TB-endemic countries in 2020 may result in hundreds of thousands of additional TB deaths^7^. Most microbiologic TB assays use culture or nucleic acid amplification to detect *Mycobacterium tuberculosis* (*Mtb*) bacilli in respiratory and extrapulmonary samples. However, young children frequently require invasive procedures to obtain respiratory samples that may contain few *Mtb* bacilli (paucibacillary TB), and often present with disseminated or extrapulmonary TB missed by respiratory sampling strategies. *Mtb* culture, the diagnostic gold standard, is slow (2–8 weeks) and detects only 30–62% of paediatric TB (ref. ^8^). PCR-based Xpert MTB/RIF (Xpert) and Xpert Ultra can provide diagnostic results in a few hours but detect only a fraction of the *Mtb*-positive sputum (65%) and gastric samples (73%) detected by *Mtb* culture^9^. Xpert and Xpert Ultra may thus detect only 20~40% of all paediatric TB cases, and their sensitivity may be even lower in children living with HIV, who may have higher rates of culture-negative TB. Also, Xpert cannot distinguish DNA derived from viable and non-viable *Mtb* bacilli, reducing its utility for the evaluation of treatment responses^10^.

Moreover, conventional immunoassays exhibit poor diagnostic performance because *Mtb*-derived factors may circulate at low concentration, exhibit poor specificity or are subject to masking effects. Also, they do not reliably distinguish latent TB infection from TB disease^11,12^. We have reported that mass-spectrometry-coupled immunoassays can improve the diagnostic performance of such biomarkers, but these approaches may have restricted utility owing to equipment and technical-expertise requirements^13–15^. Nanoparticle-enhanced EV immunoassays (NEIs) that are read by dark-field microscopes (DMFs) found in many research and clinical laboratories can be used for the ultrasensitive detection of cancer-associated extracellular vesicle (EV) biomarkers in serum, but these approaches require manual searches to identify fields of interest, making them prone to operator error and bias^16^.

**Fig. 1:**
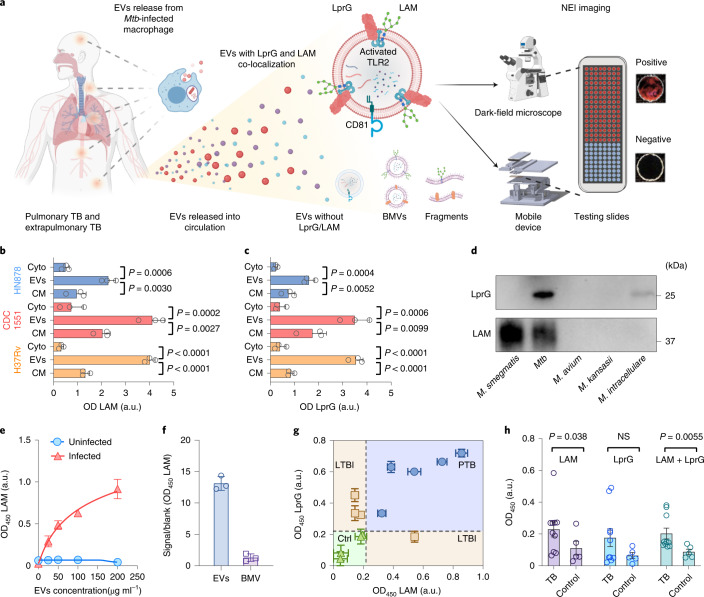
EV LAM and LprG expression as a biomarker for TB diagnosis. **a**, Rationale and assay schematic. Created with BioRender.com. **b**,**c**, Western blot densitometry of LAM (**b**) and LprG (**c**) expression in cytosolic (Cyto), EV and cell membrane (CM) factions of macrophage cultures infected with the indicated *Mtb* strains. Data indicate mean ± s.d., *n* = 3 biologically independent repeats; *P* values obtained by one-way ANOVA with Dunnett’s post-test. a.u., arbitrary units. **d**, Western blot analysis of LAM ﻿and LprG from CFP samples of patients with sequence-confirmed *M. smegmatis*, *Mtb*, *M. avium*, *M. intracellulare* and *M. kansasii* infections. **e**,**f**, EV-ELISA for LAM on EVs from *Mtb*-infected macrophages (**e**) and BMVs isolated from *Mtb* culture medium (**f**) by ultracentrifugation when captured by host EV-specific antibody. **g**, EV-ELISA for LAM and LprG on serum EVs of non-human primates with pulmonary TB (PTB), LTBI or their healthy controls (Ctrl) (mean ± s.d. of three technical replicates). **h**, EV-ELISA for LAM, LprG and integrated LAM and LprG (LAM+LprG) expression on isolated EVs from serum of children with TB (*N* = 10) and without evidence of TB (Ctrl; *N* = 5). Mean ± s.e.m., *P* values obtained by two-sided Mann-Whitney U test.

In this study, we show that automated NEI and machine learning can be used for the detection of EVs secreted by *Mtb*-infected cells (*Mtb* EVs), by leveraging the surface expression of factors abundantly expressed on the *Mtb* membrane. The NEI used in this study targeted the glycolipid lipoarabinomannan (LAM), a major component of the mycobacterial cell wall that accounts for up to 15% of *Mtb* biomass and that regulates *Mtb* virulence, as well as the membrane protein LprG, which is required for LAM distribution to the outer cell envelope^17–19^. Multiple studies have evaluated the potential of urine LAM assays for the diagnosis of TB, but few have examined serum or plasma LAM assays^20,21^, and none appear to have examined the TB diagnosis potential of serum or urine LprG assays. We hypothesized that EV LAM and LprG expression might serve as a serum biomarker of pulmonary and extrapulmonary TB since EVs accumulate in serum and can carry factors derived from *Mtb*-infected macrophages^22^ (Fig. 1a). We therefore confirmed the EV surface expression of LAM and LprG using EVs from *Mtb*-infected macrophages, investigated the ability of an integrated LAM and LprG marker to distinguish active TB disease from latent TB infection in a non-human primate model that permits accurate classification, and evaluated the ability of this integrated biomarker to diagnose TB in paediatric cohorts with and without HIV infection to address the effect of HIV on TB diagnosis. Our findings indicate that NEI had similar diagnostic performance for pulmonary and extrapulmonary TB and for culture-confirmed and clinically diagnosed TB cases in children with and without HIV.

## Results

### EVs secreted by *Mtb*-infected macrophages express LAM and LprG

To evaluate the diagnostic potential of EV LAM and LprG expression, we first examined EVs secreted by *Mtb*-infected macrophages. EVs isolated from macrophage cultures infected with or without *Mtb* had similar morphologies, but *Mtb*-infected macrophages secreted more and larger EVs (2.3 × 10^9^ vs 1.1 × 10^9^ EVs per ml), as determined by nanoparticle tracking analysis (Supplementary Fig. 1a–c). LAM and LprG were significantly enriched in EV versus cytosol and membrane fractions of macrophages infected with *Mtb* strains exhibiting variable growth rates and immunogenicity^23,24^, indicating their potential utility as strain-independent biomarkers of *Mtb* infection (Fig. 1b,c and Supplementary Fig. 1c). Western blot analysis of *Mtb*, *M. avium*, *M. intracellulare* and *M. kansasii*, and *M. smegmatis* culture filtrate protein (CFP) samples using our selected LAM and LprG antibodies detected species-specific signals. LAM signals were detected in *Mtb* and *M. smegmatis*, which rarely causes pulmonary infections, and strong and weak LprG signals were detected in *Mtb* and *M. intracellulare*. Thus, among the four mycobacteria that are major causes of pulmonary infection, our assay antibodies detected significant signals for both LAM and LprG signal only in *Mtb*, and thus dual detection of these factors is *Mtb*-specific (Fig. 1d). Neither of these factors was detected in EV fractions isolated from macrophages infected with bacteria responsible for common hospital infections (Supplementary Fig. 2). EV-ELISA, a gold standard for detection of EV surface factors^25^, detected a dose-dependent increase in LAM and LprG signals (Fig. 1e and Supplementary Fig. 1e) in serial dilutions of EVs isolated from *Mtb*-infected macrophage cultures by ultracentrifugation. These ultracentrifugation samples could contain both EVs and bacteria-derived membrane vesicles (BMVs) due to their similar physical properties^18,26^. However, the LAM and LprG EV-ELISAs used a host-specific EV protein for EV capture and detected no appreciable signal in BMV fractions from *Mtb* cultures (Fig. 1f and Supplementary Fig. 3), indicating the EV origin of the LAM and LprG signals detected in these samples.

The potential of EV-ELISA LAM and LprG signals to distinguish serum from TB, latent TB infection (LTBI) and healthy control groups was next evaluated using serum from non-human primates that were *Mtb* naïve or that developed LTBI or pulmonary TB following *Mtb* exposure. Non-human primate (NHP) models were employed in this analysis to allow more confident TB and LTBI classification since in patients it is difficult to distinguish LTBI cases (*Mtb*-infected without TB disease) from infections likely to progress to TB, but which lack clinical symptoms in the absence (incipient TB) or presence (subclinical TB) of radiographic abnormalities or microbiologic findings^27^. Serum EVs isolated from *Mtb*-naïve NHPs had low LAM and LprG signals (Fig. 1g and Supplementary Table 1); EVs from the LTBI group had elevated LAM or LprG expression, with most revealing elevated LprG signal alone; and EVs from the pulmonary TB group had elevated expression of both LAM and LprG, suggesting that these factors might serve a composite biomarker for TB diagnosis. However, the mechanism(s) involved in this apparent differential expression in TB and LTBI, and its functional significance, is not known and should be replicated in large, well-characterized longitudinal human LTBI and TB cohorts.

EV-ELISA analysis of EVs isolated from archived serum from a small, well-characterized case-control cohort of children (Supplementary Tables 2 and3) enrolled at the Vietnam National Lung Hospital (NLH) as pilot of a large, population-based TB surveillance study found that mean LAM and integrated LAM and LprG signals, but not LprG signal, differed between the TB and non-TB groups (Fig. 1h). EV-ELISAs were conducted to analyse additional factors that were reported to be abundantly secreted, membrane-associated and/or able to distinguish TB and LTBI cases^28,29^, and which had available specific antibodies. Only LAM and LprG revealed differential expression on serum EVs from TB versus non-TB cases, potentially due to low or absent EV surface expression of the additional selected factors (Supplementary Fig. 4).

### NEI sensitively detects LAM and LprG expression on *Mtb* EVs

LAM and LprG EV-ELISAs did not detect differences between the NLH TB and non-TB groups when used to directly analyse their serum (Supplementary Fig. 5), unlike results obtained with EVs enriched from these samples, demonstrating that EV-ELISA lacks the sensitivity required for a direct serum assay. We therefore investigated the feasibility of using NEIs to directly capture EVs from serum and sensitively detect gold nanorod (AuNR) probes bound to LAM or LprG on these EVs by high- or low-magnification dark-field microscope (DFM) image analysis, each of which has advantages and disadvantages. High-magnification analyses allow ultrasensitive detection but require manual focusing to detect plasmonic signals from interacting nanoparticles, which can introduce selection bias due to sampling of limited areas of the assay wells^16,30,31^. Low-magnification DFM analyses can be automated and are thus more suitable for clinical applications, but are subject to artefacts that can increase background noise and reduce sensitivity^30,32^.

**Fig. 2:**
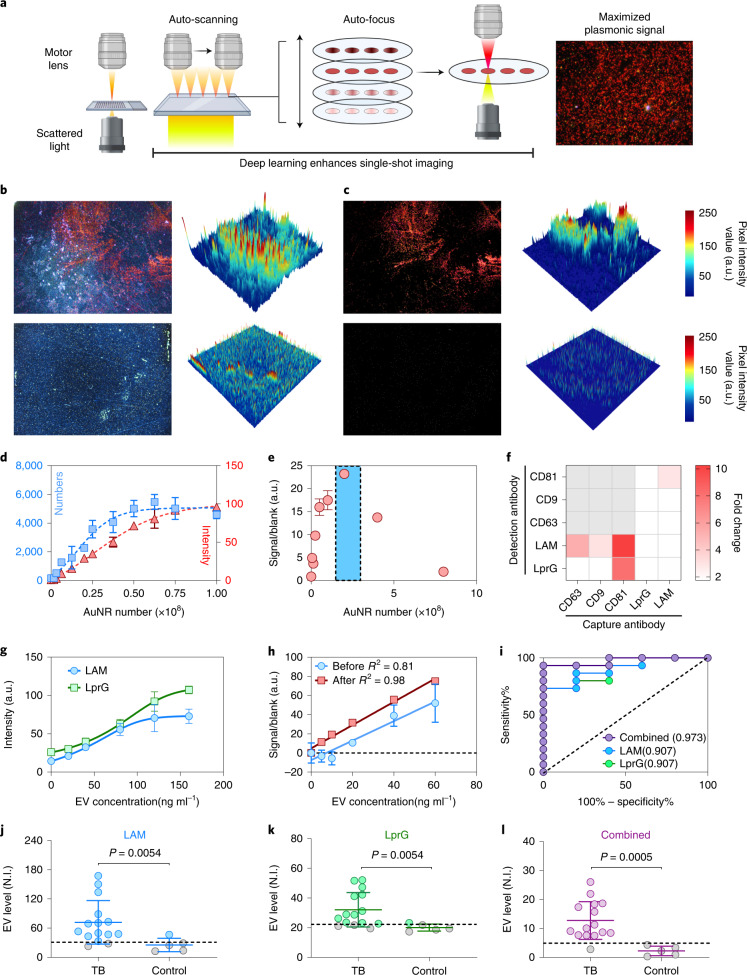
NEI optimization for sensitive detection of EV-associated TB biomarkers. **a**, Schematic of the NEI image capture workflow and signal. Created with BioRender.com. **b**,**c**, NEI images (left) and 3D heat maps (right) before (**b**) and after (**c**) image processing to remove artefacts (blue and white signal) to improve detection of AuNRs (red signal) bound to *Mtb* EVs (top) and negative control EVs (bottom). **d**, Standard curves for AuNR dilutions quantified by counting positive pixels versus mean pixel intensity. **e**, AuNR titration to maximize signal-to-noise (highlighted in blue shadow) in samples near the EV-ELISA limit of detection (150 ng EV protein per ml). **f**, NEI signal increase versus background (signal-to-noise) when using the indicated capture and detection antibody pairs to detect NEI signal from a low-concentration EV sample (150 ng protein per ml) and a negative control (blank) sample. **g**, EV LAM and LprG NEI signal linearity with an *Mtb* EV concentration curve generated using EVs from *Mtb*-infected macrophages. **h**, Linear regression line and correlation coefficient of NEI EV LAM signal without and after noise reduction in background-normalized serum samples spiked with EVs isolated from *Mtb*-infected macrophages. For **d**–**h**: mean ± s.d.; *N* = 3. **i**, Receiver operating characteristic (ROC) analysis for the ability of single and integrated (combined) EV LAM and LprG NEI signals to distinguish children with and without TB, indicating areas under the ROC curve. **j**–**l**, NEI signal for LAM (**j**), LprG (**k**) and integrated LAM and LprG (**l**) expression on serum EVs of children with TB (*N* = 15) and with no evidence of TB (*N* = 5), N.I., NEI signal intensity (arbitrary units; a.u.). Solid lines indicate mean ± s.d.; dashed lines indicate the threshold for positive signal determined in corresponding ROC analysis in **i**. *P* values were determined by two-sided Mann-Whitney U test.

We therefore established an NEI workflow (Fig. 2a) to permit automatic capture of low-magnification DFM images that were then processed with a custom noise-reduction algorithm to reduce artefacts from serum aggregates, particulates and surface scratches introduced during the assay. This algorithm detected and cropped the region of interest (ROI), converted each image into HSB (hue, saturation and brightness) colour space, and normalized and applied thresholds to each HSB channel to identify AuNR-derived signals (Supplementary Fig. 6). This markedly reduced signal artefacts, as indicated by the enhancement of AuNR signals in processed images and their pixel intensity maps (Fig. 2b,c). For signal detection, analysis of an AuNR dilution curve by quantifying positive pixels and mean pixel intensity found that the latter approach had greater linearity with less variation (Fig. 2d).

EVs isolated from *Mtb*-infected macrophages were hybridized with dilutions of anti-LAM antibody-conjugated AuNRs to determine the probe concentration required to maximize the signal-to-noise ratio at low target EV concentrations (Fig. 2e). Subsequent evaluation of capture and detection antibody pairs determined that anti-CD81 capture antibody and two anti-LAM (A194-01) and anti-LprG (Clone B) detection antibodies produced the best signal-to-noise ratios (10.21 and 7.25, respectively; Fig. 2f and Supplementary Fig. 7), and good linearity and variability over a broad EV concentration range (0–150 ng EV protein per ml; Fig. 2g). Target EVs spiked into pooled human serum were detected with high sensitivity (5 ng ml^−1^), linearity (*R*^2^ = 0.98) and variability (mean coefficient of variation (c.v.) = 4.6%, 2.1–5.3%) using the optimized NEI and analysis workflow, but with reduced sensitivity (40 ng ml^−1^), linearity (*R*^2^ = 0.81) and reproducibility (mean c.v. = 65.2%, 20.3–152.9%) when analysed without image processing (Fig. 2h). No LAM or LprG NEI signal was detected from BMVs isolated from *Mtb* culture medium (Supplementary Fig. 8). Major EV signal decreases were detected in spiked samples stored at 4 °C or ambient temperature, presumably due to EV lysis or biomarker proteolysis, but not in cryopreserved samples (Supplementary Fig. 9).

NEI analysis of serum from a small discovery cohort found that serum EV LprG and LAM signals had similar ability to distinguish the TB and non-TB groups, but that EV LAM and LprG signals integrated via a logistic model (see Methods) had superior performance (Fig. 2i), as indicated by the ability of thresholds derived from each analysis to differentiate these groups (Fig. 2i–l). Substantial signal overlap occurred between these groups near the LAM or LprG signal thresholds, resulting in three false negatives and one false positive, while the integrated LAM and LprG threshold produced a single false negative. The NEI LAM and LprG signal was detected in EV-enriched but not in EV-depleted serum fractions, confirming the EV-specificity of the NEI signal (Supplementary Fig. 10). However, the NEI signal did not differ when paediatric TB cases were stratified by age, sex or TB manifestation (pulmonary or extrapulmonary) (Supplementary Fig. 11), although there was a tendency for higher NEI signal in patients with extrapulmonary TB, which might reflect reduced immune containment associated with this form of TB. The diagnostic performance observed in the NLH cohort was validated using serum from an ongoing paediatric TB study where patients had more detailed clinical information available for case classification (Supplementary Fig. 12 and Table 4). The NEI signal yielded diagnostic sensitivities of 88.2% (15/17) and 81.8% (9/11) for confirmed and unconfirmed TB cases, respectively, and detected one of three children in the control group with one of the criteria used for clinical TB diagnosis. Conversely, NEI had high diagnostic specificity (91.7%; 11/12) for children in the control group who lacked any NIH TB diagnosis criteria (Supplementary Fig. 13 and Table 5)^33^, suggesting that the NEI signal served as a general marker of TB disease. Serum from patients with non-TB pulmonary infections with respiratory cultures positive for common bacteria strains (Supplementary Fig. 14) were negative for LAM and LprG.

### *Mtb* EV biomarker evaluation in symptomatic children with HIV at high risk for TB

**Fig. 3:**
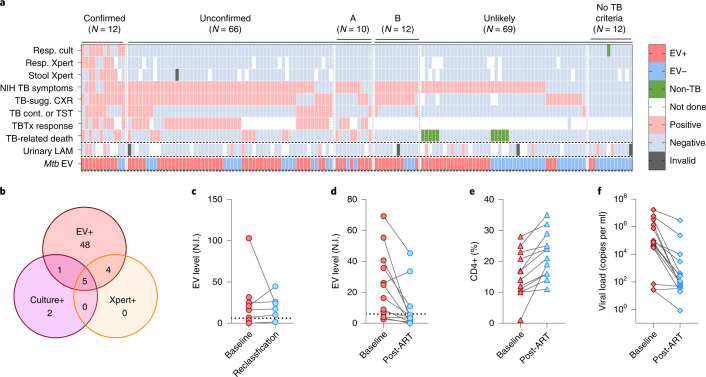
*Mtb* EV NEI diagnostic performance in children living with HIV at high risk of TB. **a**, NEI signal in children with confirmed, unconfirmed and unlikely TB as determined by positive respiratory culture/Xpert or stool Xpert results, TB-related symptoms meeting NIH criteria for the duration, chest X-ray (CXR) findings, close TB contact or positive TST, positive TBTx response, and/or TB-related death. Urine LAM results and serum *Mtb* EV results were not used for classification. Subgroup A: children reclassified from unlikely to unconfirmed TB on the basis of TBTx (TB treatment) response or TB-related death as determined by an expert review panel (see Supplementary Table 7 for criteria). Subgroup B: children reclassified from unconfirmed to unlikely TB on the basis of symptom improvement following ART initiation without TBTx initiation (alternate data for anti-TBTx response). **b**, TB cases classified by NEI, *Mtb* culture and/or Xpert test results. **c**, Positive baseline NEI signal predicts subsequent TB reclassification in children with unlikely TB assignments at enrolment (Subgroup A in **a**) who were reclassified to unconfirmed TB by investigators blinded to EV results. **d**–**f**, NEI signal decreases (**d**) among children diagnosed as unconfirmed TB cases at baseline but reclassified as unlikely TB cases due to symptom improvement without TBTx following ART initiation alone (Subgroup B in **a**), which corresponded with CD4 cell % increases (**e**) and HIV viral load reductions (**f**) following ART initiation.

We next evaluated NEI assay performance in a diagnostically challenging cohort of children living with HIV at high risk for TB-related morbidity and mortality. This analysis employed serum collected from children with HIV in the multi-site Paediatric Urgent vs post-Stabilization Highly active antiretroviral therapy (ART) initiation trial (PUSH) in Kenya who were hospitalized and not yet initiated on ART at enrolment (Supplementary Fig. 15 and Table 1). Most children in this study exhibited severe immunosuppression as well as some symptoms consistent with TB. Children were retrospectively classified on the basis of the 2015 NIH paediatric clinical TB case definitions, as confirmed TB using microbiologic evidence (positive *Mtb* culture or positive Xpert results for respiratory or stool samples); unconfirmed TB (if positive for ≥2 of the following criteria: TB-associated symptoms; abnormal TB-consistent chest radiograph (CXR); close TB exposure or evidence of *Mtb* infection (that is, positive tuberculin skin test (TST))); or positive response to TB treatment (TBTx); or unlikely TB (if they lacked two criteria for unconfirmed TB) (Supplementary Table 6). Children with confirmed and unconfirmed TB had high rates of symptoms (75.0% vs 84.9%) and CXR findings (91.7% vs 84.9%) consistent with TB, with most having positive findings for both symptoms and CXR findings (66.6% vs 71.2%; Fig. 3a), although a subgroup was reclassified from unlikely to unconfirmed TB on the basis of TBTx response or TB-related death as determined by an expert review panel (Supplementary Table 7 and Fig. 3a, subgroup A). Children classified as ‘unlikely TB’ frequently had symptoms or CXR findings consistent with TB (66.7% vs 30.2%), but a subset positive for both criteria was classified as unlikely TB due to symptom improvement following ART without TBTx initiation (Fig. 3a, subgroup B).

**Table 1:** Baseline characteristics of HIV-positive hospitalized ART-naïve children at high risk for TB evaluated by TB exosome assays for TB diagnosis (147 children)

Characteristics	Number of children with results	Total, 147 children	With confirmed TB, 12 children	With unconfirmed TB, 66 children	With unlikely TB, 69 children
Age, years	147	1.8 (0.8, 4.4)	2.9 (1.2, 6.7)	1.9 (1.1, 4.8)	1.6 (0.7, 3.9)
Female, number	147	70 (47.6)	5 (41.7)	30 (45.5)	35 (50.7)
CD4, cell count, cells per μl	146	727.5 (317, 1,240)	487 (156, 800.5)	650 (202, 1,210)	767 (524, 1,445)
CD4, %	146	15 (9, 22)	13 (6, 21.3)	12.9 (6, 19)	17 (12, 24)
**Number of children**					
With severe immunosuppression for HIV^a^	146	102 (69.9)	9 (75.0)	50 (76.9)	43 (62.3)
Wasted^b^	114	72 (63.2)	7 (87.5)	34 (66.7)	31 (56.4)
Underweight (WAZ < –2)	147	95 (64.6)	8 (66.7)	47 (71.2)	40 (58.0)
With signs or symptoms suggestive of TB^c^	147	111 (75.5)	9 (75.0)	56 (84.9)	46 (66.7)
With TST ≥ 5 mm	134	7 (5.2)	1 (9.1)	5 (8.5)	1 (1.6)
With TB exposure	147	25 (17.0)	7 (58.3)	15 (22.7)	3 (4.4)
With CXR suggestive of TB	141	86 (61.0)	11 (91.7)	56 (84.9)	19 (30.2)
With positive respiratory *Mtb* culture or Xpert^d^	147	11 (7.5)	11 (91.7)	0	0
With Stool Xpert positive^e^	130	7 (5.4)	7 (63.6)	0	0
With urine LAM positive^f^	116	12 (10.3)	3 (37.5)	3 (5.8)	6 (10.7)
With TBTx initiated	147	57 (38.8)	10 (83.3)^g^	46 (69.7)	1 (1.5)
With TBTx response	57	47 (82.5)	7 (70.0)	40 (87.0)	0
Dead^h^	147	29 (19.7)	5 (41.7)	14 (21.2)	10 (14.5)

The numbers are provided as medians, with interquartile ranges or the percentage of participants with positive results (with respect to the number of children with results) given in brackets. WAZ, weight-for-age *z* score; WHZ, weight-for-height *z* score.

^a^WHO age-specified percentage of CD4 cut-offs for severe immunosuppression (age < 12 months, <25%; age 12–35 months, <20%; age ≥36 months, <15%) or, in the absence of CD4-percentage data, CD4 count (age < 12 months: <1,500 cells per μl; age 12–35 months, <750 cells per μl; age ≥ 36 months, <350 cells per μl).

^b^Among children 5 years and under: WHZ < –2 or mid-upper arm circumference (MUAC) < 12.5 cm.

^c^Persistent cough (>14 d), fever (>7 d), failure to thrive, or lethargy (>7 d). Failure to thrive corresponds to wasted (WHZ < –2 or MUAC < 12.5) or underweight (WAZ < –2) at enrolment (growth trajectories unavailable before enrolment).

^d^Sputum or gastric aspirate.

^e^Includes one child with unconfirmed TB who had indeterminate Stool Xpert results.

^f^Includes one child with unconfirmed TB and 2 children with unlikely TB who had invalid urine LAM results. Colour change corresponding to manufacturer reference card grade ≥1 was considered positive at the time of the study.

^g^Two children with confirmed TB died before initiating TB treatment.

^h^Within 6 months of enrolment.

NEI analyses performed on PUSH cohort serum by operators blinded to clinical information and using positive signal thresholds determined in the NLH discovery cohort detected confirmed TB and unconfirmed TB with 83.3% and 76.1% diagnostic sensitivity (Table 2). NEI results had similar sensitivity for unconfirmed TB cases with and without clinical TB diagnoses (Table 2), and detected all but 2 confirmed TB cases (Fig. 3b). NEI results also detected the majority (54.4%) of children with unlikely TB who had at least one criterion required for unconfirmed TB diagnosis (Table 2). Since an age-matched low-risk cohort was not available, NEI diagnostic specificity was estimated in a subgroup of children with unlikely TB who lacked all NIH criteria for unconfirmed TB. Median *Mtb* EV signal in this group was significantly lower than in all other groups. Positive TBTx response rates were high in children with confirmed and unconfirmed TB (87.0% vs 70.0%; Table 1). Mortality after TBTx initiation was higher in children with confirmed vs unconfirmed TB (30.0% vs 8.7%; Table 2), but even higher (50.0%) in children with unconfirmed TB who were not diagnosed or treated during the study. Mortality was lower in children with unlikely TB who had no vs any TB criteria (0% vs 17.9%), although children with unlikely TB who died frequently had missing microbiologic or CXR results (Fig. 3a). Urine LAM tests performed during the study had poor diagnostic sensitivity for confirmed (37.5%; 3 of 8) and unconfirmed (5.8%; 3 of 52) TB cases with valid test results, and moderate specificity for unlikely TB cases (88.9%; 48 of 54), including children with no TB criteria (81.8%; 9 of 11).

**Table 2:** Diagnostic performance of *Mtb* EV for TB among HIV-positive hospitalized ART-naïve children at high risk for TB (147 children^*^)

	Confirmed TB^a^	Unconfirmed TB^a^ (66 children)		Unlikely TB^a^ (69 children)	
	Microbiological confirmation (12 children)	Clinical TB diagnosis^b^ (46 children)	No clinical TB diagnosis^b^ (20 children)	Any NIH criteria^c^ (57 children)	No NIH criteria^c^ (12 children)
Number of children positive according to *Mtb* EV	10	35	13	31	2
Sensitivity, %	83.3 (51.6, 97.9)	76.1 (61.2, 87.4)	65.0 (40.8, 84.6)	54.4 (40.7, 67.6)	–
Specificity, %	–	–	–	45.6 (32.4, 59.3)	83.3 (51.6, 97.9)
TB-untreated deaths, % and fraction	100%, 2/2	–	50%, 10/20	17.9%, 10/56	0%, 0/12
TB-treated deaths, % and fraction	30.0%, 3/10	8.7%, 4/46	–	0%, 0/1	–
*Mtb* EV levels, median	24.6 (6.9, 66.5)	16.6 (6.9, 31.6)	8.5 (3.9, 18.6)	7.1 (1.9, 23.8)	2.7 (0.6, 4.6)
*P* value^d^	0.001	0.0005	0.01	0.02	Reference

Confidence intervals (95%) for the sensitivities and specificities, and interquartile ranges for the *Mtb* EV levels are given in brackets.

^*^Includes 137 children with serum analysed at baseline; 7 at time of ‘unconfirmed TB diagnosis’ (6 at 2 weeks and 1 at 4 weeks post enrolment); and 3 ‘unlikely TB’ cases with missing baseline serum who had serum analysed at 2 weeks post enrolment.

^a^Assessed post-hoc on the basis of international consensus clinical-case definitions^33^: paediatric TB, children who were prospectively diagnosed during the study period by clinical staff and who received TBTx during the study period; no TB diagnosis or TBTx during study, children who were not clinically diagnosed and did not receive TBTx during the study period; NIH TB criteria, children who had at least one of the two diagnostic criteria required for TB diagnosis by the NIH algorithm.

^b^Assessed during the study. Clinical TB diagnosis: initiated TBTx. No clinical TB diagnosis: did not initiate TBTx.

^c^Positive CXR or TB exposure within the last two years; or TST ≥ 5 mm; or NIH TB symptoms: persistent cough (>14 d), fever (>7 d), failure to thrive, or lethargy (>7 d). Failure to thrive corresponds to wasted (WHZ < –2 or MUAC < 12.5) or underweight (WAZ < –2) at enrolment.

^d^Wilcoxon rank-sum test (two-sided), compared to ‘unlikely TB with no TB symptoms’ and ‘negative CXR’.

### Serum *Mtb* EV signal correspondence with TB reclassifications and treatment

Most children with unconfirmed TB were diagnosed using enrolment data, but a few were reclassified from unlikely to unconfirmed TB (15.1%; 10/66) on the basis of subsequent findings (Supplementary Table 8). Reclassification decisions were made without knowledge of EV results, but most unlikely to unconfirmed TB reclassifications (80%; 8/10) occurred in children who had detectable *Mtb* EV NEI signal at baseline, and this signal remained positive in children who had sera available after reclassification (Fig. 3c). Conversely, all unconfirmed to unlikely TB reclassifications occurred in children who had symptom improvement without TBTx initiation, most of whom had positive *Mtb* EV NEI signal at baseline (75%; 9/12) that decreased by the time of reclassification (88.9%; 8/9; Fig. 3d). Most of these children had HIV viral load and CD4+ T-cell count improvements following ART initiation (Fig. 3e,f), suggesting that improved immune function may have contained nascent TB cases. Median NEI signal also markedly decreased from pre- to post-TBTx initiation (median 5.5 months post initiation) in confirmed and unconfirmed TB cases (Table 3). Taken together, these findings suggest that decreases in serum *Mtb* EV NEI signal were associated with resolution of TB disease in response to TBTx and, perhaps, *Mtb* containment or clearance during immune reconstitution.

**Table 3:** Median *Mtb* EV levels at TB diagnosis and post initiation of TB treatment

	Number of children	Pre-TBTx initiation^a^	Post-TBTx initiation^a^	*P* value^b^
All TB cases	50	15.1 (7.5, 37.4)	4.9 (1.5, 13.4)	<0.0001
Confirmed TB	9	35.1 (9.1, 64.2)	5.3 (0.9, 13.5)	0.0056
Unconfirmed TB	41	14.0 (6.9, 35.1)	4.7 (2.1, 13.1)	0.0015

IQRs are given in brackets.

^a^Median 5.5 months (IQR; 3.1, 5.7) between TBTx initiation and post-TBTx initiation.

^b^Paired Wilcoxon signed-rank test (two-sided).

### Design and validation of a portable DFM device for *Mtb* EV assay signal readout

**Fig. 4:**
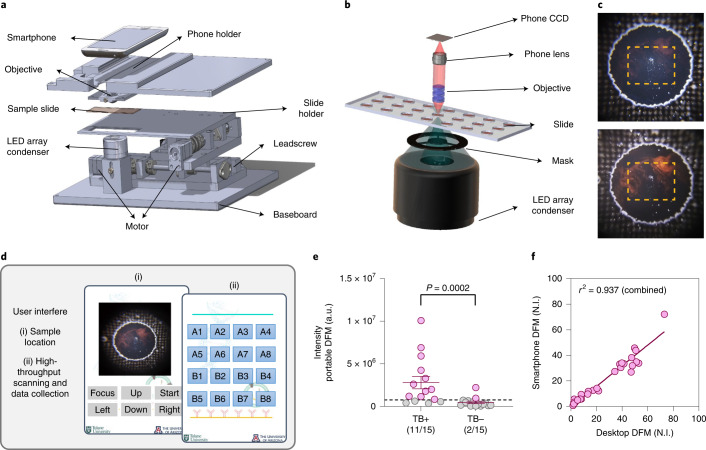
Design and performance of a smartphone-based NEI point-of-care TB diagnosis approach. **a**–**c**, Schematic of a portable smartphone-based DFM device for NEI assay readout (**a**) and its dark-field condenser mask (**b**), and the effect of this mask on NEI AuNR signal (red) intensity of the targeted *Mtb* EVs from images (**c**) collected without (top) and with (bottom) the condenser mask. **d**, Schematic of smartphone app menu workflow. **e**, EV NEI signal from serum samples of children with TB (*N* = 15) and with no evidence of TB (*N* = 15). Data indicate mean ± s.e.m.; dashed line indicates the TB detection threshold for TB positive (red) or TB negative (grey) sample assignment; *P* values were determined by two-sided Mann-Whitney U test. **f**, Comparison of integrated EV LAM and LprG NEI signals obtained for the samples in **e** using a desktop DFM and the portable smartphone DFM device, indicating the linear regression line and squared Pearson correlation coefficient.

To adapt this assay method for resource-limited settings where TB is prevalent, we employed a three-dimensional (3D) printer to fabricate an inexpensive and portable smartphone-based DFM device that can scan a 144-well assay slide in 5 min (Fig. 4a and Supplementary Table 9). This device employed an aluminum slide holder to add stability and maintain focus during automated scanning, and an illumination mask to block stray light and improve DFM signal intensity (Fig. 4b,c). A smartphone app developed for this device allowed manual centering and focusing of the first slide well, after which the app automatically centred, focused and captured images of the remaining wells, saving all images to separate files for download and analysis (Fig. 4d). This device produced results similar to benchtop DFM results when employed to read NEI data, accurately identifying 73% (11/15) of TB cases and 87% (13/15) of non-TB cases (Fig. 4e). Normalized NEI signal intensities for integrated and single biomarker signals were also similar between the portable and benchtop approaches (Fig. 4f and Supplementary Fig. 16).

## Discussion

We have evaluated the utility of adopting an NEI approach to rapidly detect *Mtb* EV biomarkers in small serum volumes for TB diagnosis, particularly in children with HIV—a population that is often missed by sputum-based diagnostics. Refining a standard NEI approach permitted automated image capture and the ultrasensitive detection of the target signal. The assay captured EVs from serum, eliminating the common EV-immunoassay requirement for purified EV samples, which are normally isolated by methods that involve trade-offs between time, labour, expense and EV yield, purity and integrity that limit their clinical feasibility. The NEI workflow is similar to that employed by ELISAs and may be suitable for use in clinical-laboratory settings after further assay optimization and clinical-validation studies. Because there are currently no widely accepted EV biomarkers of TB, in this study we evaluated the potential diagnostic utility of measuring serum levels of EVs expressing two *Mtb*-derived factors associated with TB virulence—LAM and LprG. We found that the EV expression of these markers was enhanced in TB cases, and that multiplex detection of these two factors could differentiate LTBI and TB in an NHP model. LAM and LprG are both highly expressed in *Mtb* bacilli, which could contribute to their diagnostic performance. However, other abundant *Mtb*-derived factors identified as potential targets by a literature search did not exhibit similar capacity to distinguish TB and non-TB cases, including the membrane protein Ag85b, a mycolyl transferase required for efficient biosynthesis of the *Mtb* cell wall. In this analysis, it is not clear whether the secreted *Mtb* proteins analysed in this study (CFP10, ESAT6, MPT51 and MPT64) form stable membrane interactions that would permit their detection on serum EVs.

NEI analysis of an NHP model of LTBI and TB indicated that serum EV expression of both LAM and LprG was required to distinguish TB from LTBI. LAM is a virulence factor that is expressed on the *Mtb* cell wall, where it can bind to the macrophage mannose receptor to facilitate cellular entry of *Mtb* bacilli into host phagocytes, inhibit phagosome-lysosome fusion and modulate the immune response to promote continued intracellular survival of *Mtb* required for TB development^19,34–36^. LprG plays an essential role in the localization of LAM to the outer cell envelope of *Mtb* bacilli, and LprG null *Mtb* mutants exhibit reduced LAM surface expression and virulence, decreased *Mtb* entry into host macrophages, reduced biogenesis and/or integrity of the *Mtb* cell envelope, failure to inhibit phagosome-lysosome fusion and reduced intracellular replication rates. LprG is thus essential for LAM activity, as LprG deficiency attenuates *Mtb* virulence without altering LAM expression. LprG expression might thus be expected to be downregulated in NHPs with LTBI cases; however, our results suggest the opposite case: downregulation of LAM and upregulation of LprG expression on serum EVs from NHPs diagnosed with LTBI, whereas both markers were elevated on serum EVs from NHPs diagnosed with TB. Several mechanisms could explain this finding, including the downregulation of LAM expression or the inhibition of LprG activity to limit LAM transport, both of which would be expected to limit LAM expression on the *Mtb* membrane and, presumably, LAM expression of EVs secreted by *Mtb*-infected cells during LTBI. However, the mechanisms responsible for this differential expression and its functional significance are unclear and merit further study, including replication in larger, well-defined LTBI and TB cohorts with less diagnostic uncertainty.

NEI analysis of *Mtb* EVs exhibited good diagnostic sensitivity for both confirmed and unconfirmed TB in a diagnostically challenging population of children living with HIV, including children not clinically diagnosed during the parent study despite extensive TB work-up. Notably, the NEI assay identified children with HIV who are often missed by sputum-based assays and diagnosed by physician judgement and non-specific findings. NEI diagnostic sensitivity in this group is of particular relevance because mortality was more than five times higher in untreated children who were not diagnosed with TB during clinical evaluation than those who were diagnosed and treated at evaluation, in keeping with reported high mortality rates in children who do not receive TBTx due to missed diagnoses. Notably, serum from several children exhibited positive *Mtb* EV signal before their TB diagnosis by clinical findings, suggesting that serum *Mtb* EV signal has potential as a means for early TB diagnosis, which is of particular importance in this population since a third of the children identified in NEI analysis died at or shortly after their diagnosis by conventional means. Most PUSH cohort children had valid urine LAM assay results, but these had poor diagnostic sensitivity for confirmed and unconfirmed TB (37.5% and 5.8%) compared with NEI (90.1% and 72.5%), although both assays showed similar diagnostic specificity (83.3% and 81.8% for NEI and urine LAM) for children with no TB criteria. There are several possible explanations for these differences. First, blood is homoeostatic unlike urine and may thus be less subject to dilution and degradation effects that could influence urine LAM detection. Specifically, serum EV LAM should achieve a steady state that balances secretion and lysis, phagocytosis and secretion rates in a constant volume, whereas urine LAM degradation and dilution effects can be influenced by variable urination intervals and dilution or concentration effects. Second, soluble LAM and EV LAM degradation rates may differ. Finally, urine LAM tests detect soluble LAM whereas EV LAM NEIs detect EV-bound LAM, hence the two methods are not directly comparable, although this question could be addressed in future studies.

NEI *Mtb* EV signal markedly decreased following TBTx initiation for both confirmed and unconfirmed TB cases, in agreement with TB symptom improvement, suggesting that *Mtb* EV level might be useful as a surrogate for TBTx response. Similar decreases were also observed in a subset of children who met the criteria for unconfirmed TB when evaluated by their baseline data, but who were reclassified as unlikely TB due to improvement of their TB suggestive symptoms following ART initiation without TBTx initiation. NEI *Mtb* EV signal decreases in this context suggest that the children may have had nascent TB which was at least partially contained by improved immune function following ART initiation. Quantifiable serum biomarker results may also serve as a surrogate when evaluating TBTx response, particularly for extrapulmonary TB cases which are not amenable to repeat sampling. This is a critical TB management need and the monitoring ability of NEI should thus be evaluated in studies designed and powered to address this question.

NEI *Mtb* EV signal was also detected in several children diagnosed with unlikely TB who had one of two algorithmic features required for unconfirmed TB classification. Median serum *Mtb* EV signal did not markedly differ between these groups, suggesting that a substantial fraction of the unlikely TB cases may have had nascent TB that was not diagnosed within the study evaluation period. NEI signal was markedly lower in the unlikely TB group that lacked a single positive result for any of the criteria required for TB diagnosis, with only two children having an EV signal above the established threshold for TB diagnosis (83% specificity). However, all these children were at high risk for TB and thus do not represent a clear non-TB control population. Specificity estimates for this study were thus compromised by the absence of a large non-symptomatic population that could more confidently be characterized as non-TB cases.

This study has limitations. First, we employed cryopreserved repository samples collected in different studies, although our results indicate that short-term sample-handling differences should not affect target EV signal. Second, although all children enrolled in the PUSH cohort underwent extensive TB evaluation and had at least two microbial test results, it is possible that missing data could have affected case classifications made using the modified NIH TB classification criteria. Furthermore, not all the PUSH cohort children who received TBTx had equivalent longitudinal serum samples available for analysis, preventing a detailed treatment-response study following TBTx initiation. Larger prospective studies are warranted to confirm and extend the results in children and adults with and without HIV, with pulmonary and extrapulmonary TB disease, and with appropriate negative controls. However, despite the cohort-size limitations, TB diagnosis rates were similar in all three paediatric cohorts (Table 4), suggesting that HIV infection and severe immunosuppression did not degrade NEI diagnostic performance. NEI diagnosis rates and mean signal also did not markedly differ among children with and without HIV who had pulmonary or extrapulmonary TB. Thus, serum *Mtb* EV detection may be particularly valuable for TB diagnosis in young children, people with HIV and individuals with extrapulmonary TB, who can be difficult to diagnose using conventional methods and samples. Third, both LAM and its carrier protein LprG are required for normal cell-wall biogenesis in mycobacteria, but at least one antibody is reported to recognize a LAM epitope that is specific for *Mtb* complex species^37^. No studies appear to have analysed the potential of LprG for TB diagnosis, but although several non-tuberculous mycobacteria (NTM) express LprG orthologues, those from the two NTM species responsible for most NTM respiratory infections—*M. avium* and *M. intracellulare*—have limited sequence identity with Mtb LprG. Finally, we analysed LAM and LprG expression on EVs, but BMVs could also serve as potential biomarkers for TB diagnosis^18,26^. A similar NEI assay to detect these BMVs directly from serum would require validation of a capture antibody that permits direct isolation of BMVs present in serum, and this needs to be addressed in future studies designed for this purpose.

**Table 4:** Performance metrics for the diagnosis of children with TB, for the three cohorts and for the pooled datasets

Cohort	Performance metric	Confirmed TB	Unconfirmed TB	Unlikely TB with any criteria	No NIH criteria and non-TB control
NLH (20 children)	Fraction of children	14/15	–	–	0/5
	Sensitivity, %	93.3 (68.1, 99.8)	–	–	–
	Specificity, %	–	–	–	100.0 (47.8, 100.0)
DR (43 children)	Fraction of children	15/17	9/11	1/3	1/12
	Sensitivity, %	88.2 (63.6, 98.5)	81.8 (48.2, 97.7)	33.3 (0.8, 90.6)	–
	Specificity, %	–	–	66.7 (9.4, 99.2)	91.7 (61.5, 99.8)
PUSH (147 children)	Fraction of children	10/12	48/66	31/57	2/12
	Sensitivity, %	83.3 (51.6, 97.9)	72.7 (60.4, 83.0)	54.4 (40.7, 67.6)	–
	Specificity, %	–	–	45.6 (32.4, 59.3)	83.3 (51.6, 97.9)
Pooled (210 children)	Fraction of children	39/44	57/77	32/60	3/29
	Sensitivity, %	88.6 (75.4, 96.2)	74.0 (62.8, 83.4)	53.3 (40.0, 66.3)	–
	Specificity, %	–	–	46.7 (33.7, 60.0)	89.7 (72.6, 97.8)

The fraction of children refers to the fractions that were positive according to *Mtb* EV. Confidence intervals (95%) for the sensitivities and specificities are given in brackets.

Our current assay is suitable for use at sites with minimal laboratory equipment, but still requires blood collection and separation, and multiple pipetting and wash steps before being read via a microscope or a smartphone device. Efforts are ongoing to refine the assay for use in point-of-care settings that lack the resources to perform it, including adapting the assay to a lateral-flow format that could use fingerstick blood samples and be read by either a modified version of our existing smartphone device, or perhaps by direct inspection. This would also allow prospective real-time evaluation of an inexpensive portable device suitable for use in resource-limited settings, which could enhance testing options in regions with high endemic TB rates that account for 95% of new TB cases and mortality^4,38^.

## Methods

### EV isolation and characterization

*Mtb* and cell culture and BMVs isolation are described in the supplementary method as previously reported^39^. Cryopreserved serum aliquots (1 ml) that were processed for EV isolation were rapidly thawed in a 37 °C water bath, vortexed for 3 s, then 300 μl aliquots were transferred to 2 ml centrifugation tubes, mixed with 1.2 ml PBS, and then centrifuged at 1,200 r.p.m. for 15 min at 4 °C to pellet large particulates and debris. Supernatants were centrifuged at 2,000 *g* for 30 min at 4 °C, passed through a 0.45 μm filter, centrifuged twice at 10,000 r.p.m. and 4 °C for 45 min, and then at 110,000 *g* and 4 °C for 90 min. Resulting EV pellets were suspended in 1 ml PBS, centrifuged at 110,000 *g* and 4 °C for 3 h, and then suspended in 50 μl PBS and characterized by bicinchoninic acid assay and NanoSight nanoparticle tracking analysis (Malvern Panalytical; 5 μg ml^−1^ with 5 replicates) to determine protein content and EV concentration and size distribution. Cell culture supernatants corresponding to the same number of H37Rv-infected macrophages and uninfected macrophages were concentrated for EV analysis by centrifugation at 4 °C and 3,000 r.p.m. for 15 min using 50 ml 10 kDa Copolymer Styrene ultrafiltration tubes (Millipore Sigma). This procedure concentrated 200 ml of culture supernatant to 100 μl. Concentrated samples were processed for EV isolation and resulting EV suspensions were quantified by NanoSight nanoparticle tracking analysis at the same dilution factor to compare the number of EVs secreted by H37Rv-infected macrophages and uninfected macrophages. EV concentrations obtained represented the area under the curve of the number of particles detected over the entire analysed size range, as reported by the NanoSight NS 300 instrument. For transmission electron microscope analyses, carbon-coated Cu grids (400 mesh) were incubated for 30 s at room temperature with 5 μl EV aliquots containing 1 mg ml^−1^ protein, blotted with filter paper to remove excess liquid, incubated with 2 μl 1% osmium tetroxide for 2 min at room temperature to stain membranes, blotted to remove this solution, and imaged at the indicated magnification using a Tecani F30 microscope (FEI Company) with an accelerating voltage of 200 kV. All EV samples isolated in this study were aliquoted and stored at –80 °C until use, rapidly thawed in a 37 °C water bath and immediately processed for EV assay or other analysis.

### EV-ELISA analyses

Standard EV capture plates used for EV-ELISA were generated by adding 100 µl aliquots of mouse antibody specific for human CD81 (BioLegend) to each well of 96-well microtitre plates (5 µg antibody per ml in PBS) and incubating these plates for 16 h at room temperature. Wells were then washed 3× with 260 µl PBST (30 s per wash), then blocked by incubation with 250 µl blocking buffer (1% w/v BSA in PBST) for 2 h at 37 °C, and again washed 3× with PBST. To analyse optimal EV capture conditions for NEI, wells were alternately incubated with 100 µl aliquots of mouse antibodies (5 µg ml^−1^) specific for EV surface markers including CD9, CD63, CD81 and different clones of anti-LAM/LprG antibodies (1 μg ml^−1^).

For EV-ELISAs, each well was incubated overnight at 4 °C with 100 µl dilutions of isolated EVs or serum, washed 3× with PBST and then incubated with 100 µl PBST containing 1 μg ml^−1^ of the indicated detection antibodies, including monoclonal antibodies specific for *Mtb* LAM, LprG, LpqH, MPT64, CFP10, ESAT6, MPT51, MPT32 and KatG and polyclonal antibodies specific for *Mtb* Ag85b and Ag85c, for 1 h at 37 °C, washed 3× with PBST, then incubated with 100 µl PBST containing 0.5 μg ml^−1^ HRP-labelled goat anti-mouse/rabbit/human IgG (Jackson ImmunoResearch) for 30 min at 37 °C, and washed 3× with PBST. After the final wash step, wells were incubated with 50 µl TMB (Sigma) for 10 min at room temperature and then mixed with 50 µl of 2 M H_2_SO_4_ to stop the reaction, after which target EV for each well were read using a microplate reader (Tecan, Ultra) to measure optical density (OD)_450_ values after subtracting OD_650_ signal.

### NHP *Mtb* infection and sample collection

No live Indian-origin rhesus macaques were used in this study. Cryopreserved NHP plasma analysed in this study was obtained from archived material from NHPs infected with *Mtb* in previously reported studies (approved by the respective Institutional Animal Care and Use Committee as well as the Institutional Biosafety Committee at Tulane National Primate Research Center and Southwest National Primate Research Center)^40–42^. Briefly, specific-pathogen-free, retrovirus-free, mycobacteria-naive, adult rhesus macaques were assigned to three experimental groups that received different *Mtb* exposures (none, low and high). Samples for the negative control (*Mtb* naïve) cohort were obtained from four uninfected rhesus macaques that were not exposed to *Mtb* during the study period. Samples for the LTBI cohort were obtained from four rhesus macaques subjected to a low-dose *Mtb* aerosol exposure event (~10 c.f.u. of *Mtb* CDC1551), which had positive results for TST within a month after exposure but did not exhibit any signs of TB, instead maintaining asymptomatic LTBI-like infections throughout the study (~22 weeks). Samples for the TB cohort were obtained from five rhesus macaques that were subjected to a high-dose *Mtb* aerosol event (~200 c.f.u. of *Mtb* CDC1551), which developed active TB disease characterized by weight loss, pyrexia, elevated serum C-reactive protein levels, elevated chest radiograph scores consistent with TB, detectable *Mtb* c.f.u. levels in bronchoalveolar lavage fluid, higher lung bacterial burden and associated lung pathology at the study endpoint. Lung tissue collected at the study endpoint was randomly sampled by pathologists blinded to animal treatment using a grid, as described previously^41,42^.

### NEI analyses

EV capture slides used for NEI analyses were generated by adding 1 µl aliquots of mouse antibody specific for human CD81 EV capture or indicated antibodies (5 µg ml^−1^) to each position of a 144-well mask affixed to a microscope slide and incubating these slides for 16 h at 4 °C. All incubation steps in this analysis were performed in a humidified chamber to reduce evaporation effects. Following capture antibody binding, slides were washed 3× with PBST, blocked with 1 µl per well SuperBlock (PBS) blocking buffer (Cytiva) for 1 h at 4 °C and then washed 3× with PBST before incubation with 1 µl of serum or isolated EV samples. Cryopreserved serum aliquots were stored at –80 °C until use, rapidly thawed in a 37 °C water bath and centrifuged at 10,000 *g* for 20 min to remove large debris. Serum or EV samples were incubated on EV capture slides for 16 h at 4 °C, washed 3× with PBS, hybridized for 1 h at 37 °C with 1 µl of the specified biotinylated detection antibody (1 μg ml^−1^), washed 3× with PBS and incubated for 1 h at 37 °C with neutravidin-functionalized AuNR (Nanopartz) at the indicated concentrations. After the AuNR incubation step, slides were washed once with PBST and distilled water to remove unbound particles before they were subjected to DFM photography (NIS-Elements AR 5.11) and image analysis (MATLAB 2020a and ImageJ 1.53). To evaluate the linearity of the NEI assay response and calculate its estimated limit of detection, an EV standard curve was generated by spiking healthy human serum with an EV fraction generated by ultracentrifugation of pooled supernatants of *Mtb*-infected macrophage cultures to produce a high concentration EV standard (60 ng ml^−1^) that was then used to generate 40, 20, 10 and 5 ng ml^−1^ EV dilutions using the same healthy human serum sample. A linear regression model with a single explanatory variable was used to calculate the slope and correlation coefficient (*R*^2^) of the NEI curves generated before and after image correction of the same samples. Coefficients of variation were calculated from triplicates of each concentration standard to determine the range of c.v. and averaged to determine the mean c.v. of the assay measurements. We determined the sensitivity of the assay to be the concentration of the lowest EV serial dilution standards that produced signal that significantly differed from the blank assay standards, as evaluated using Student’s *t*-test.

### DFM noise-reduction algorithm

NEI signal was analysed using a custom algorithm that first identified the area of each assay to be processed and then subtracted DFM artefacts and background noise. This algorithm detected the high-intensity boundary of each well and calculated the centre position of the circular region formed by its high-intensity boundary. The central area of this region was selected for analysis to avoid potential ‘coffee-ring effects’ caused by the accumulation of residual AuNRs at well edges after the final wash step. To remove DFM artefacts and background, images were converted to HSB colour space and the values in each channel from the 16-bit images were normalized to a 0 to 1 range since hue is measured in degrees (0° to 360°). The hue and saturation channels were employed to identify AuNR signals and remove artefacts using a set of training images from slides coated with pure AuNRs and mixtures of AuNRs and human serum. Hue was employed to identify pixels that matched the AuNR scattering range and saturation was employed to evaluate the colour intensity (purity) of pixels that matched the AuNR range. Pixels with hue channel values outside the red scattering range of the AuNR signal (≥0.8 and ≤0.05 on the hue colour wheel) were excluded, as were pixels with very low saturation values (≤0.05). AuNR signal in processed images was measured using MATLAB (2020a) with the parameters described above.

### Clinical cohort evaluations

No statistical methods were used to pre-determine sample size, which was determined by the number of patients with sample available for each retrospective cohort.

### Dominican Republic (DR) cohort

Specimens and associated clinical data were collected from 48 children aged ≤18 years who were enrolled and consecutively followed at the Robert Reid Children’s Hospital or the Hugo Mendoza Children’s Hospital, using a protocol approved by the University Dominicana O&M Institutional Review Board (IRB), which included an amendment for the analyses performed in this study. Children were seen by the attending clinicians at these institutions, who obtained written informed consent from the parent or legal guardian. Children who were considered to be TB cases fulfilled the diagnostic criteria established for the Classification of Intrathoracic Tuberculosis in Children. Confirmed TB cases were defined by positive *Mtb* culture and/or Xpert results for pulmonary or extrapulmonary specimens. Children were classified as unconfirmed TB cases if they met the acceptance thresholds for at least two of the following criteria: TB-associated symptoms, a TB-consistent abnormal CXR, a positive TST result or a known TB exposure, and/or a positive TBTx response. TB cases with HIV infection were not excluded from this cohort. Non-TB controls who lacked any evidence of TB disease or infection were age-matched to and enrolled from the same neighbourhoods as the TB cases. Children enrolled in the non-TB control group who subsequently had positive TST results were excluded and replaced by enrolment of another age-matched control that met the enrolment criteria for this group.

### NLH cohort

Specimens and associated clinical data were collected from 20 children aged ≤17 years who consecutively visited the NLH in Ha Noi, Vietnam for clinical assessment and medical evaluation, using a protocol approved by the NLH IRB, which included an amendment approving the analyses performed in this study. Children were excluded from participation if they were >17 years of age. All NLH cohort children were evaluated by clinicians in the NLH Department of Pediatrics and were enrolled only after written informed consent was provided by a parent or legal guardian. Children were excluded if they had laboratory-documented anaemia (haemoglobin < 9 mg dl^−1^) or if informed consent was not obtained for all study procedures, but were not excluded on the basis of evidence of HIV infection or ART. All TB cases were defined by positive *Mtb* culture and/or Xpert results for pulmonary or extrapulmonary specimens. Children enrolled as controls had negative QuantiFERON-TB Gold Plus (QFT), Xpert and *Mtb* culture results, had TB ruled out by clinical assessment by experienced paediatric TB specialists and received broncho-pulmonary diagnoses other than TB.

### PUSH cohort

The PUSH study was a randomized controlled trial (NCT02063880) performed in Kenya to evaluate whether urgent (<48 h) vs post-stabilization (7–14 d) ART intervention improved survival in hospitalized HIV-infected children aged <12 years^43^. This protocol was approved by the University of Nairobi Institutional Review Board and included an amendment for the analyses performed in this study. At enrolment, children were systematically screened for TB symptoms and TB exposure and underwent intensive TB evaluation, including CXR, sputum or gastric aspirates for Xpert and culture, urine for LAM antigen testing, and stool for Xpert, irrespective of TB symptoms^44^. Serum was collected and cryopreserved at enrolment and at 2, 4, 12 and 24 weeks post enrolment. CXRs were read by a radiologist using standardized reporting forms to identify findings suggestive of TB developed by the South African Tuberculosis Vaccine Initiative^33^. Diagnostic results were available to study clinicians and TBTx was initiated at their discretion on the basis of Kenyan guidelines^45^.

Children were post-hoc categorized as confirmed TB (positive *Mtb* culture or Xpert result from a respiratory or stool sample), unconfirmed TB (≥2 of the following criteria: TB-associated symptoms, TB-consistent abnormal CXR, positive TST result (induration ≥ 5 mm) or known TB exposure, and/or TBTx response) or unlikely TB (not meeting other criteria) on the basis of NIH international consensus clinical-case definitions for paediatric TB (Supplementary Table 6)^33^. TB symptoms were defined as a cough lasting >2 weeks, weight loss/failure to thrive, fever lasting >1 week, and/or lethargy lasting >1 week. For children who died during the study, an expert panel reviewed cases and came to a consensus regarding whether death was considered to be likely, possibly, or unlikely related to TB. Death considered likely or possibly related to TB was employed as an additional criterion for unconfirmed TB classification (Supplementary Table 7). For the assay evaluation, children with unconfirmed TB were further stratified by whether or not they received a clinical TB diagnosis and started TBTx during the study. Children categorized as unlikely TB were further stratified by the presence or absence of features employed for unconfirmed TB diagnosis. Children with cryopreserved serum available within 2 weeks of TB diagnosis, including within 2 weeks of TBTx initiation, were evaluated to estimate TB diagnostic performance of *Mtb* EV NEI results. Children who initiated TBTx with samples within 2 weeks of TBTx initiation and at least one additional subsequent sample were eligible for the treatment-response analyses.

Participant characteristics were summarized by frequency and proportion for categorical variables, and by median and interquartile range (IQR) for continuous variables (Table 1). NEI diagnostic performance for TB diagnosis was estimated using 95% confidence intervals assuming a binomial distribution stratified by NIH paediatric TB clinical-case definitions (Table 2). Many children in the unlikely TB category either had CXR suggestive of TB or had TB symptoms (but not meeting unconfirmed TB criteria); therefore, we further stratified the unlikely TB category by the presence or absence of abnormal CXR and TB symptoms to identify a potentially more appropriate negative control group.

CXR results were extracted from the hospital medical records for 14 of the 24 children with missing CXR data (4 confirmed, 2 unconfirmed and 18 unlikely) and abnormalities consistent with TB identified using notations based on South African Tuberculosis Vaccine Initiative (SATVI) CXR classification criteria. Median *Mtb* NEI EV levels were evaluated by Wilcoxon rank-sum test compared to the reference of unlikely TB with no TB symptoms and negative CXR. For treatment response, median *Mtb* NEI EV levels at TBTx initiation and at latest available sample were evaluated by paired Wilcoxon signed-rank test (median time between TBTx initiation and latest available sample 5.5 months (IQR 3.1–5.7)).

### Portable DFM device

The mobile DFM slide scanning system (270 × 190 × 106 mm) has a slide scanning range of 82 mm × 38 mm and consists of a mechanical scanning system with two-step motors, a dark-field light source, an interchangeable smartphone with a miniaturized objective, an IOIO-OTG board (DEV-13613, SparkFun Electronics) and two motor driver boards (ROB-12779, SparkFun Electronics) to control the step motors and communicate with the smartphone component via Bluetooth. This system employs two lithium batteries: a 7.6 V 3,500 mAh Lipo battery (3.35 × 1.97 × 0.55 inches, GAONENG) supplies power for the electronics, and an 11.1 V 3,200 mAh Lipo battery (5.16 × 1.73 × 0.67 inches, HOOVO) supplies power for the motors. Most of the additional parts were printed with a commercial 3D printer (Objet 30 prime, Stratasys), but the slide holder employed to ensure the stability of the slide during the scanning process was fabricated from an aluminum plate using a computer numerical control milling machine. The dark-field light source consists of an integrated dark-field condenser containing an illumination numerical aperture (NA) ranging from 0.7 to 0.9 and an array of 3 mm white LEDs with a viewing angle of 30 °, and employs a mask placed in front of the condenser to block stray light and improve the contrast of dark-field images. The objective consists of three identical doublets; it has a focal length of 3.4 mm, NA of 0.25, working distance of 1 mm and field of view of 1.6 × 1.6 mm. The camera of the mobile phone component of this device, a Moto G6, had a 3.95 mm focal length, a working F-number of 1.8 and a sensor with 12-megapixel resolution (3,072 × 4,096 pixels, pixel size 1.4 μm).

### Statistical analysis

GraphPad Prism (version 9.0), Microsoft Office 365 Excel, SAS OnDemand version 9.4 for Academics (https://welcome.oda.sas.com/login)﻿ and Stata 17.0 were employed to generate figures and heat maps, and perform statistical analyses. Potential differences between groups were analysed by two-sided parametric or non-parametric (Kruskal-Wallis) one-way analysis of variance (ANOVA) with multiple comparison test, Wilcoxon signed-rank test, Student’s *t*-test or Mann-Whitney U test. Data distributions were assumed to be normal for parametric tests but was not formally tested. All other data met the assumptions of these statistical tests. Logistic regression was used to test whether the combination of LAM and LprG could predict TB diagnosis and to determine the optimal weights of each marker (https://stats.blue/Stats_Suite/logistic_regression_calculator.html)^46^. Statistical significance was evaluated by comparing the area under the curve (AUC) values generated by LAM, LprG and the weighted sum of LAM and LprG, where accounting for the correlation leads to a larger *z* value and thus, a smaller *P* value: ${{{z}}} = \frac{{{{{\mathrm{Area}}}}_1 - {{{\mathrm{Area}}}}_2}}{{\sqrt {\mathrm{SE}_{\mathrm{Area}1}^2 + \mathrm{SE}_{\mathrm{Area}2}^2} }}$, while *α* = 2 × (1−NORMSDIST(*z*)), as determined by the Microsoft Excel NORMSDIST function^47^. Hypothesis testing was performed using a two-sided *α* of 0.05 to evaluate significant differences. Both characteristics (LAM and LprG) were entered into this logistic regression model without other covariates. Data are presented as mean ± s.d. unless noted otherwise. The investigators were not blinded to the samples analysed in the training cohort, cell culture studies and the NHP cohort, but were blinded to the identification of the DR and PUSH cohort samples. No data points were excluded from the analyses.

### Reporting summary

Further information on research design is available in the Nature Research Reporting Summary linked to this article.

## Supplementary information

Supplementary Information Supplementary methods, figures, tables and references.

Reporting Summary

## Data Availability

The data supporting the results in this study are available within the paper and its Supplementary Information. Source data are provided with this paper. The raw and analysed datasets generated during the study are too large to be publicly shared, yet they are available for research purposes from the corresponding author on reasonable request. De-identified patient data are available from the corresponding author, subject to IRB approval.

## Code availability

Source code used in this work is available for non-commercial purposes from the corresponding author on request.
