# Supplementary material for: Diagnosis of paediatric tuberculosis by optically detecting two virulence factors on extracellular vesicles in blood samples
Source: Nat Biomed Eng. 2022 Aug 19;6(8):979–91. doi: 10.1038/s41551-022-00922-1 (PMC9391224; doi:10.1038/s41551-022-00922-1)
Supplement: Supplementary file 1 — Supplementary methods, figures, tables and references. [file 41551_2022_922_MOESM1_ESM.pdf]

nature portfolio

https://doi.org/10.1038/s41551-022-00922-1

Supplementary information

Diagnosis of paediatric tuberculosis by optically detecting two virulence factors on extracellular vesicles in blood samples

In the format provided by the authors and unedited

Contents

Supplementary Methods
Supplementary Figs. 1–17
Supplementary Tables 1–9
References

Supplementary Methods

***Mtb* Culture and BMVs isolation.** *Mtb* strains H37Rv, CDC1551 and HN878 were obtained from Houston Methodist Hospital and cultured at 37°C in roller bottles to mid-log phase (OD600 = 0.5–0.6) in a pH 7.0 minimal protein-free medium containing 0.1% (v/v) glycerol, 1 g/L KH_2PO_4, 2.5 g/L Na_2HPO_4, 0.5 g/L asparagine, 50 mg/L ferric ammonium citrate, 0.5 g/L $MgSO_4 \times 7\ H_2O$, 0.5 mg/L $CaCl_2$, and 0.1 mg/ L $ZnSO_4$, with or without 0.05% tyloxapol (v/v). To harvest BMVs, *Mtb* cultures were grown in 500 mL minimal medium until mid-log phase were harvested at 4000 g for 10 min at 4°C. Supernatants were collected and passed through a 0.22 *μ*m filter twice, and resulting filtrate samples were stored at -80°C until processed to isolate BMVs as described in previous reports.[1]

THP-1 cell culture and differentiation. THP-1 monocytes were purchased from the American Type Culture Collection (ATCC; Manassas, VA) and cultured at 37 °C in a 5% CO_2 incubator in RPMI 1640 supplemented with 10% FBS. Macrophage differentiation was performed by incubating 10 T175 flasks containing ~2.5×10^7 viable THP-1 monocytes/flask (5~7×10^5 cells/mL; ≥ 95% viability) for 24 with 325 nM PMA (Sigma Aldrich, USA), after which the flasks were washed 3× with 37°C PBS to remove PMA and non-adherent cells before culturing the adherent, differentiated THP-1 macrophages in RPMI 1640 supplemented with 10% FBS.

For experiments using bacteria-infected macrophages, mid-log phase 10 mL cultures of *Mtb* H37Rv, CDC1551, or HN878, or cultures of *E. coli*, *S. aureus*, *P. aeruginosa*, *S. pneumonia*, *K. pneumonia* were pelleted by centrifugation at 3000 g for 10 min at 4°C, and resulting bacterial pellets were suspended in 10 mL of RPMI 1640 / 10% FBS without penicillin and streptomycin, de-clumped using a brief sonication step, and passed 10 times through syringe fitted with 27-gauge needle (VWR, Norm-Ject, USA). *Mtb* suspensions were then mixed with an additional 10 mL of antibiotic-free RPMI 1640 / 10% FBS, and 0.1 mL aliquots of suspensions were added to T175 flasks containing ~2.5×10^7 differentiated THP-1 macrophages cultured in 20 mL antibiotic-free RPMI 1640/10% FBS to obtain a multiplicity of infection (MOI) of 10. After 4 h incubation, cell cultures were washed 3× with 37°C PBS to remove extracellular *Mtb* bacilli and cultured in RPMI 1640 without FBS for 48h, after which culture supernatant was passed through 0.22um filters to remove *Mtb* bacilli and generate samples for EV and soluble protein analyses. Culture filtrates were stored at -80 °C while aliquots were inoculated into mycobacterial growth indicator tubes and assessed for *Mtb* growth after 3-4 weeks of culture to confirm the absence of viable *Mtb* bacilli remained in these samples. Cultured macrophages were recovered by trypsin digestion and split into samples that were analyzed for viability and employed to generate cell lysates for Western blot analysis of target proteins.

For experiments using culture filtrate protein (CFP), aliquots containing 100 µg CFP were added to T175 flasks containing ~2.5×10^7 differentiated THP-1 macrophages cultured in 20 mL RPMI 1640 / 10% FBS. After 4 h incubation, cell cultures were washed 3× with 37°C PBS to remove extracellular CFP and cultured in RPMI 1640 without FBS for 48h, after which culture supernatant were collected to generate samples for EV and soluble protein analyses. Cultured macrophages were recovered by trypsin digestion and split into samples that were analyzed for viability and employed to generate cell lysates for Western blot analysis of target proteins.

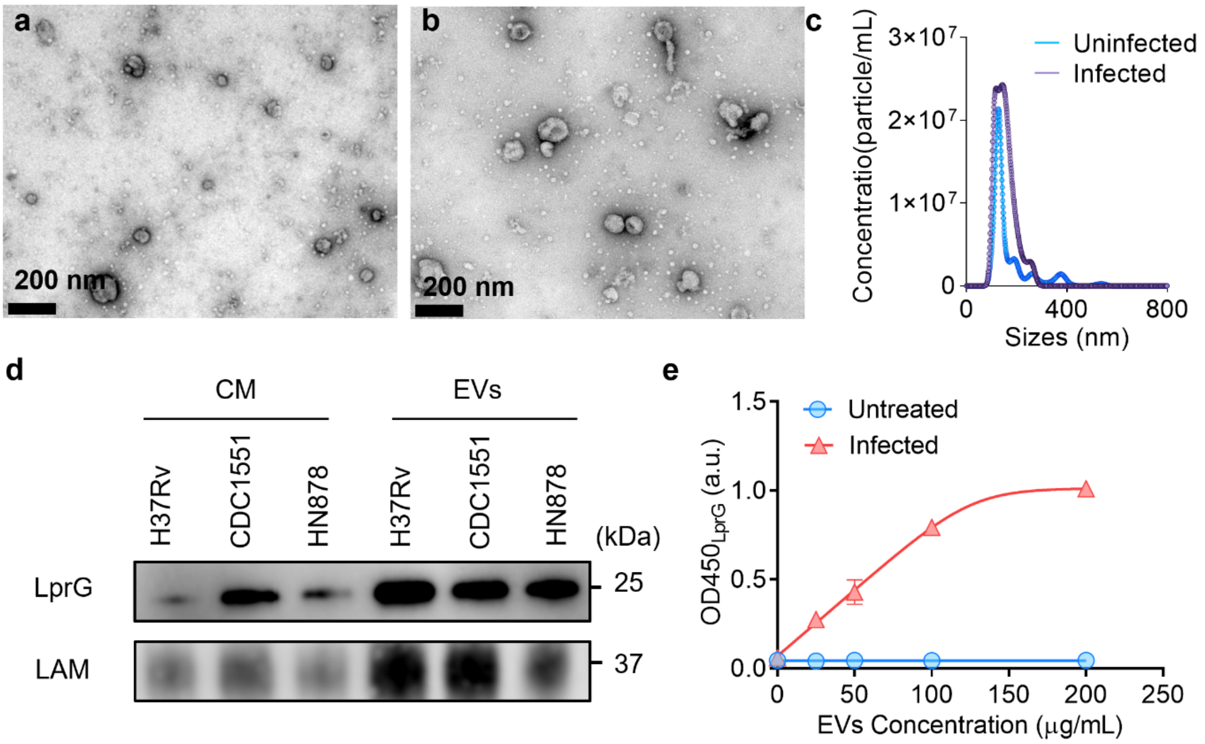

Supplementary Fig. 1 l Identification of LAM and LprG on EVs as potential marker for TB diagnosis. (**a-b**) TEM images of EVs isolated from (**a**) uninfected or (**b**) *Mtb H37Rv*-infected macrophages. (**b**) Size distribution of EVs secreted by uninfected and *Mtb* H37Rv-infected macrophages. (**d**) Western blot analysis of LAM and LprG content in cell membrane (CM) and EV fractions of macrophages infected with the indicated *Mtb* strains. (**e**) EV ELISA signal for EV surface LprG expression on increasing concentrations of EVs isolated by ultracentrifugation from the supernatants of untreated macrophages or macrophages infected with *Mtb*. Mean ± SD of three replicates per dilution. Images shown in (**a-b**) and (**d**) reflect representative data from at least 3 independent experiments that produced similar results.

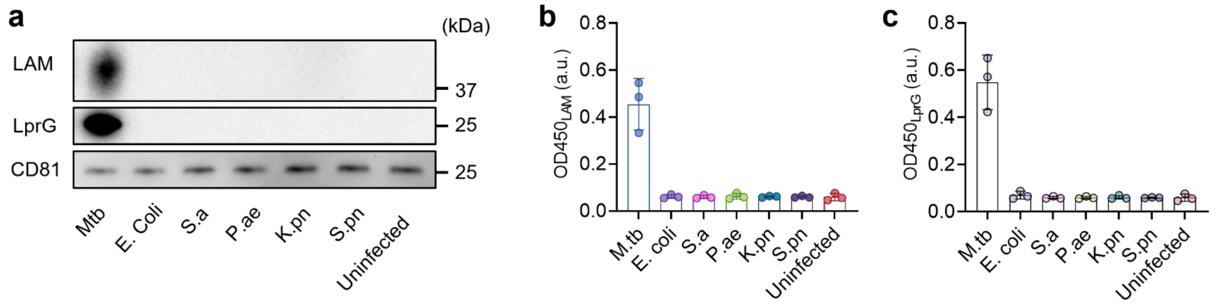

Supplementary Fig. 2 l (**a**) Western blot analysis of LAM and LprG content or (**b-c**) EV ELISA analysis of the EV surface expression of (**b**) LAM and (**c**) LprG in protein mass-equivalent EV samples isolated by ultracentrifugation from the cell culture supernatants of macrophages infected with or without *Mtb*, *Escherichia coli*, *Staphylococcus aureus*, *Pseudomonas aeruginosa*, *Streptococcus pneumonia*, *Klebsiella pneumonia*. Data indicate as Mean ± SD values of triplicate assays.

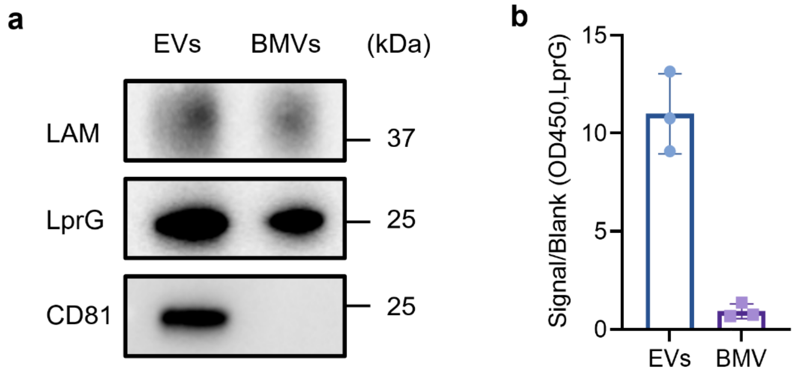

Supplementary Fig. 3 l (**a**) Western blot analysis of LAM, LprG, and CD81 in EVs isolated from the supernatants of *Mtb*-infected macrophages by ultracentrifugation or BMVs isolated from *Mtb* culture medium by ultracentrifugation. Both lanes were loaded with an equal amount of vesicle extract. (**b**) EV ELISA results for EV surface LprG signal when captured by host EVs-specific antibody. EV-depleted serum was spiked with EVs and BMVs isolated from *Mtb*-infected macrophages or *Mtb* culture after which serum EVs were isolated by ultracentrifugation prior to analysis by EV ELISA. Data indicated Mean ± SD of triplicate assays.

a

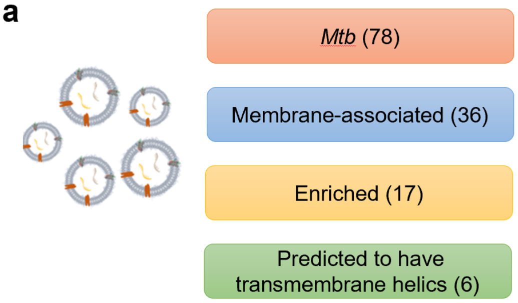

b

Accession	Description
**P9WQP1**	Ag85B
**P9WJE1**	ESX-1 secretion-associated protein EspA
**I6Y2I9**	Probable conserved integral membrane protein GN=Rv3092c
**P9WQN9**	Diacylglycerol acyltransferase/mycolyltransferase=fbpC
**P9WP07**	Uncharacterized membrane protein Rv2637
**P9WNK7**	6 kDa early secretory antigenic target GN=esxA

c

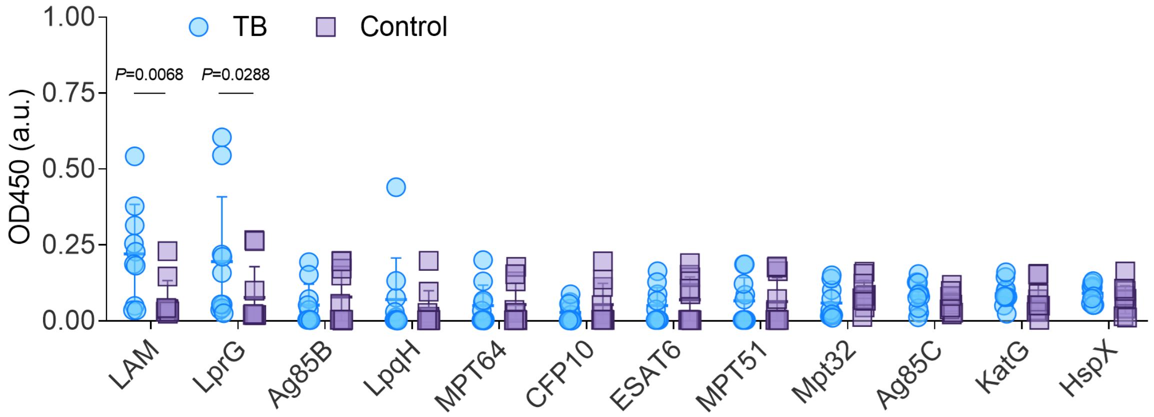

Supplementary Fig. 4 l Detection of *Mtb*-derived factors on EVs isolated from *Mtb*-infected macrophage culture supernatants and serum of TB cases. (**a**) Label-free liquid chromatography mass spectrometry (LC-MS) proteomic analysis of proteins preferentially enriched in the EV fraction vs. the EV-depleted medium from culture supernatants of *Mtb* H37Rv-infected macrophages. This analysis detected 78 *Mtb*-derived factors, 36 of which were annotated as membrane-associated, and 17 of these demonstrated significant enrichment (≥ 3-fold, p<0.05) in the EV vs. EV-depleted culture medium fractions. (**b**) Significantly enriched EV proteins with a candidate transmembrane region suggestive of their direct membrane association. (**c**) EV ELISA signal for the indicated *Mtb*-derived factors in the serum EVs of children with and without TB (N=10/group). Mean ± SD of three technical replicates per sample; p-values were determined by two-sided Mann-Whitney tests.

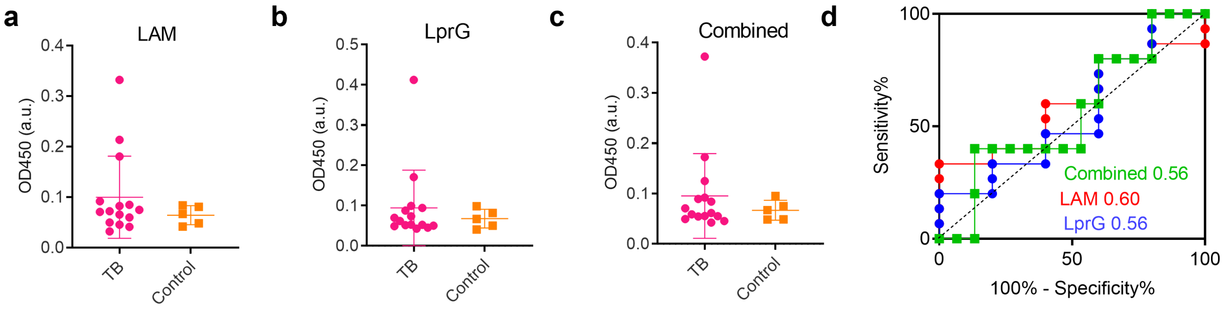

Supplementary Fig. 5 l EV ELISA signal for (**a**) LAM and (**b**) LprG, and (**c**) integrated EV ELISA LAM and LprG signal, from the serum of NLH cohort children with TB and without TB (control). Mean± SD of three technical replicates per sample. (**d**) Receiver operating characteristic (ROC) curve analyses evaluating the ability of EV LAM and EV LprG signal and their combined signal to distinguish children with and without TB using ELISA, as indicated by their area under the ROC values.

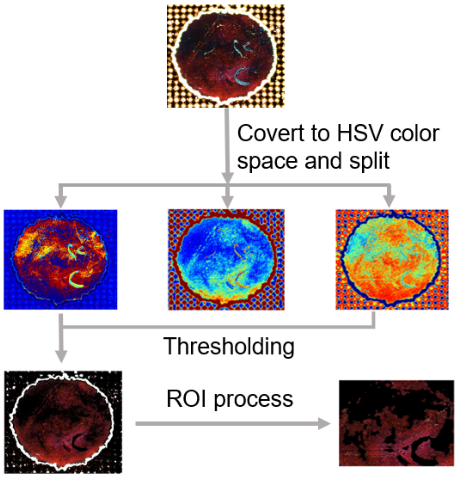

Supplementary Fig. 6 l Schematic of the procedure involved in the image processing algorithm to remove signal artefacts and background noise from NEI DFM images.

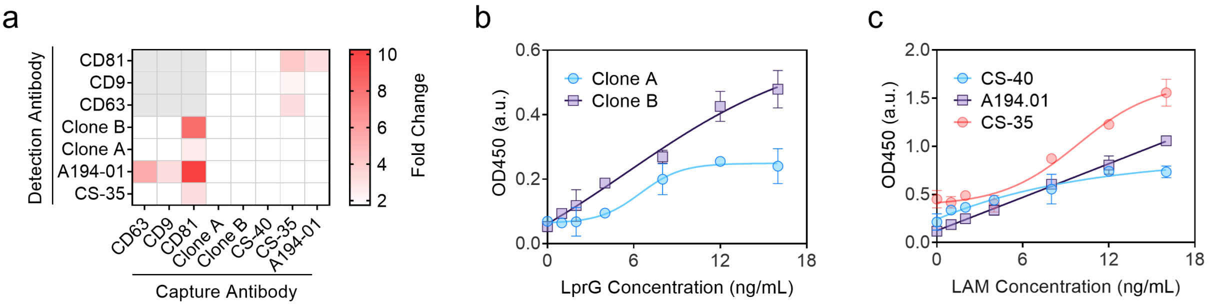

Supplementary Fig. 7 I Optimization of the NEI approach to improve high sensitivity detection of EVs carrying *Mtb*-derived factors. (**a**) Heatmap of the relative NEI AuNR signal intensity generated upon employing the indicated EV (CD9, CD63, and CD81), LAM (CS-35 and A194-01), and LprG (clone A and B) capture antibodies and detection antibodies to analyze EVs samples (150 ng protein/mL) isolated from H37Rv-infected macrophages. (**b-c**) EV ELISA signal obtained when using the indicated (**b**) LprG and (**c**) LAM detection antibodies to detect commercial LprG and LAM samples bound to the surface of the ELISA plate wells at the indicated concentrations. Mean ± SD of three technical replicates per sample.

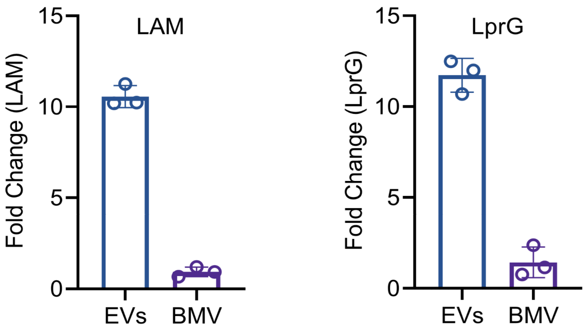

Supplementary Fig. 8 l NEI LAM and LprG signal detected in EV-depleted serum spiked with EVs and BMVs isolated from *Mtb*-infected macrophages or *Mtb* cultures after normalization to signal from serum spike with EVs isolated from uninfected macrophages. Data indicated Mean± SD of triplicate assays.

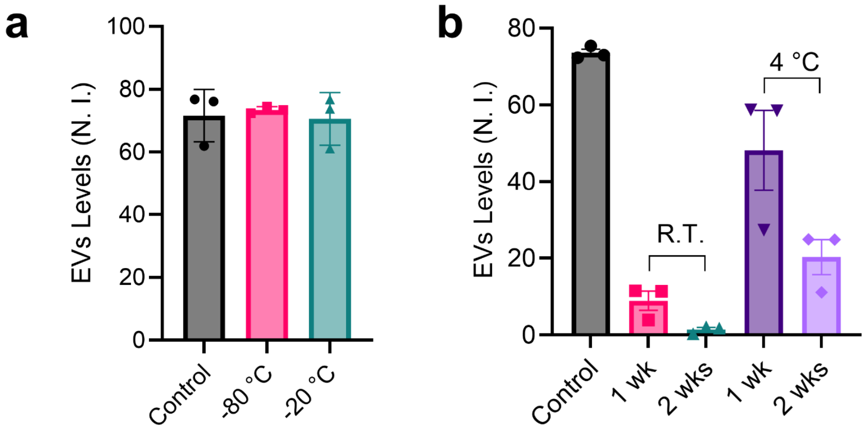

Supplementary Fig. 9 l Samples storage effects on NEI LAM signal in serum samples spiked with EVs isolated from *Mtb*-infected macrophages and then immediately analyzed (Control) or analyzed (**a**) after storage for 1 month at -20°C or -80°C or (**b**) after 1 or 2 weeks storage at room temperature (R.T.; ~22°C) or 4°C. Data indicate as Mean ± SE values of triplicate assays.

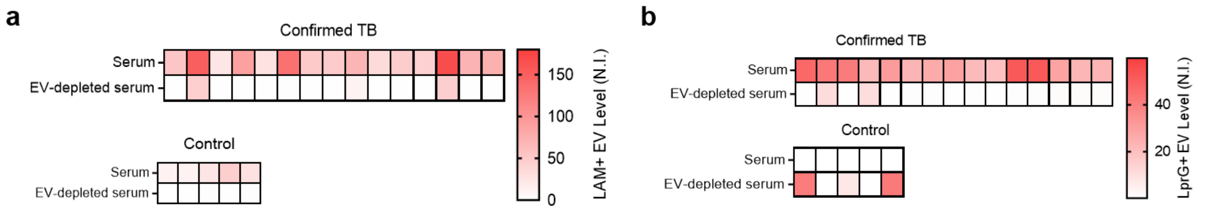

Supplementary Fig. 10 l Training cohort study for marker validation in pediatric TB. (**a-b**) NEI EV signal for (**a**) LAM or (**b**) LprG from serum and EV-depleted serum obtained from NLH cohort children with TB (N=15) and without TB (N=5).

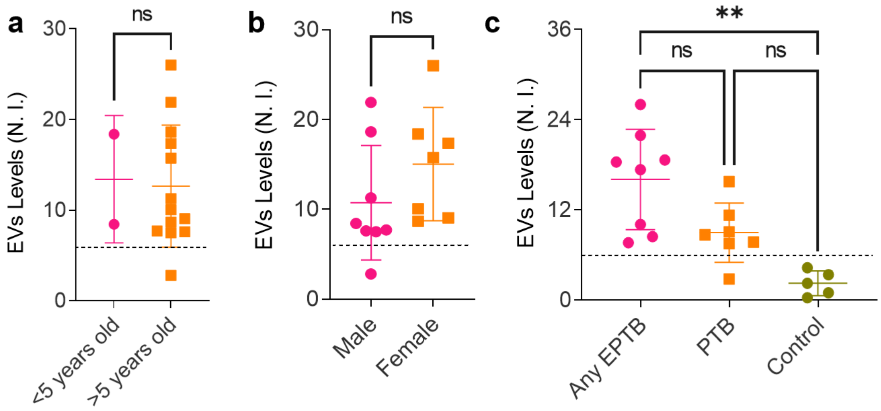

Supplementary Fig. 11 l Training cohort study for marker validation. The influence of (**a**) age, (**b**) sex, and (**c**) TB presentation (pulmonary TB [PTB] and or any extrapulmonary TB [EPTB] manifestation (EPTB only or EPTB+PTB) on NEI signal detected in serum EVs from NLH cohort children with TB (N=15). Solid lines denote the mean ± SD of each group. No significant NEI differences were identified between these groups by age or sex (ns: $p>0.05$ by two-sided Mann-Whitney U-test), and only the EPTB group differed from the non-TB control group, likely due to the limited statistical power of the analysis (ns: $p>0.05$, or * $p<0.05$ and ** $p<0.01$ by two-sided non-parametric Kruskal-Wallis one-way ANOVA with Dunn's post-test). Mean ± SD of three technical replicates per sample, dashed lines indicate the threshold for positive signal determined in corresponding ROC analysis.

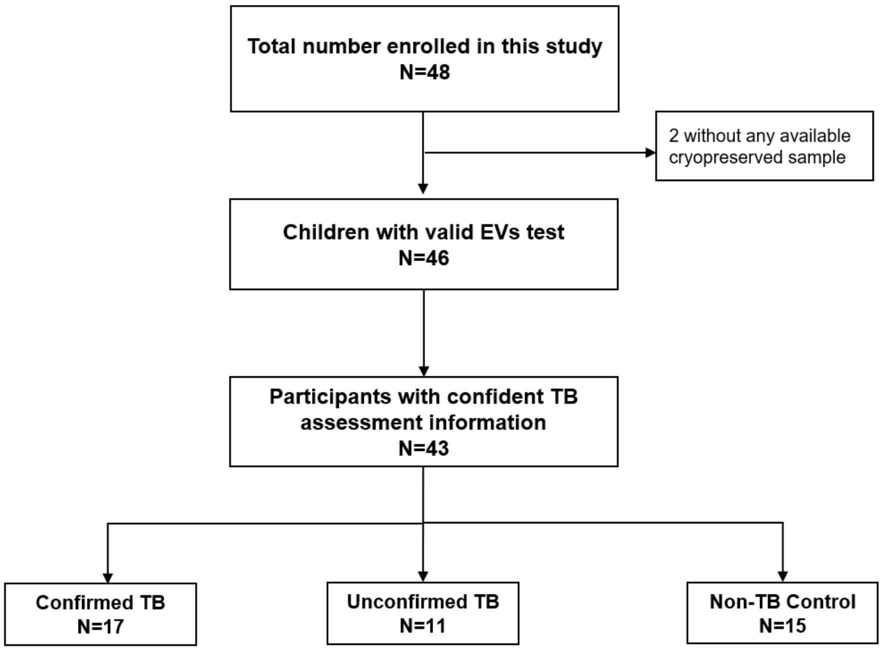

Supplementary Fig. 12 l Flow chart for inclusion and exclusion of the DR cohort children and their subgroup categorizations. Confirmed TB cases were defined by positive *Mtb* culture and/or Xpert results. Children were classified as unconfirmed TB cases if they met the acceptance thresholds for at least two of the following criteria: TB-associated symptoms, a TB-consistent abnormal CXR, had a positive TST result or a known TB exposure, and/or had a positive TB treatment response. Children who lacked any evidence of TB disease or infection, were age-matched to and enrolled from the same neighborhoods as the TB cases. Children enrolled in the non-TB control group who subsequently had positive TST results were excluded and replaced by enrollment of another age-matched control that met the enrollment criteria for this group.

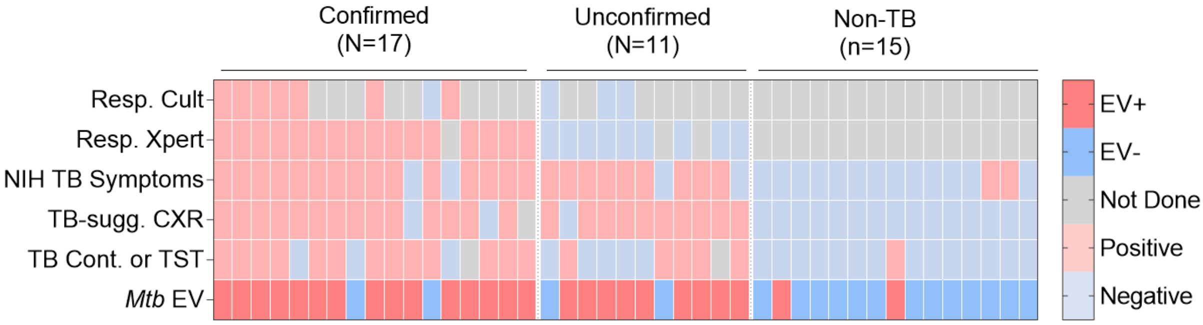

Supplementary Fig. 13 l NEI signal in DR cohort children classified into confirmed, unconfirmed, and non-TB groups, as determined by respiratory culture/Xpert or stool Xpert results, TB-related symptoms that met NIH thresholds, TB-suggestive chest X-ray (CXR) findings, close TB contact or TST results.

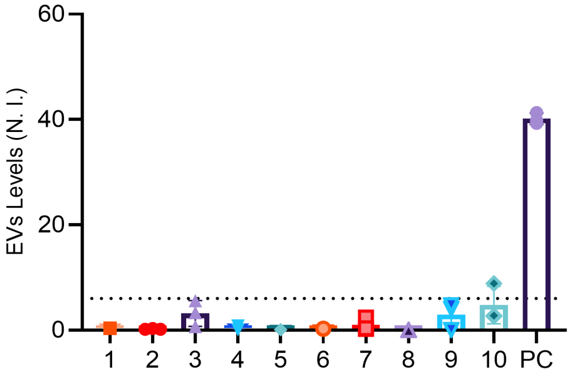

Supplementary Fig. 14 I NEI signal for integrated EV LAM and LprG expression from serum samples of 10 non-TB patients whose respiratory cultures were positive for *Pseudomonas aeruginosa* (*P.ae*; # 6,8); Methicillin-Resistant *Staphylococcus aureus* (MRSA; #2); *P.ae* and Methicillin-Sensitive *Staphylococcus aureus* (MSSA) (#1); *P.ae* and MRSA (4,9,10); *P.ae*, MRSA, *Achromobacter xylosoxidans* and *E. coli* (#5); MSSA and *S. maltophilia* (#7); or MSSA and *Haemophilus Influenzae* (#3). Data indicate the mean ± SD of 3 replicates for each sample; dashed lines indicate the threshold for positive signal determined in corresponding ROC analysis of NEI results of serum EVs from individuals in the Dominican Republic cohort classified as confirmed TB and Non-TB. TB: positive control sample representing serum from a Dominican Republic patient with confirmed TB.

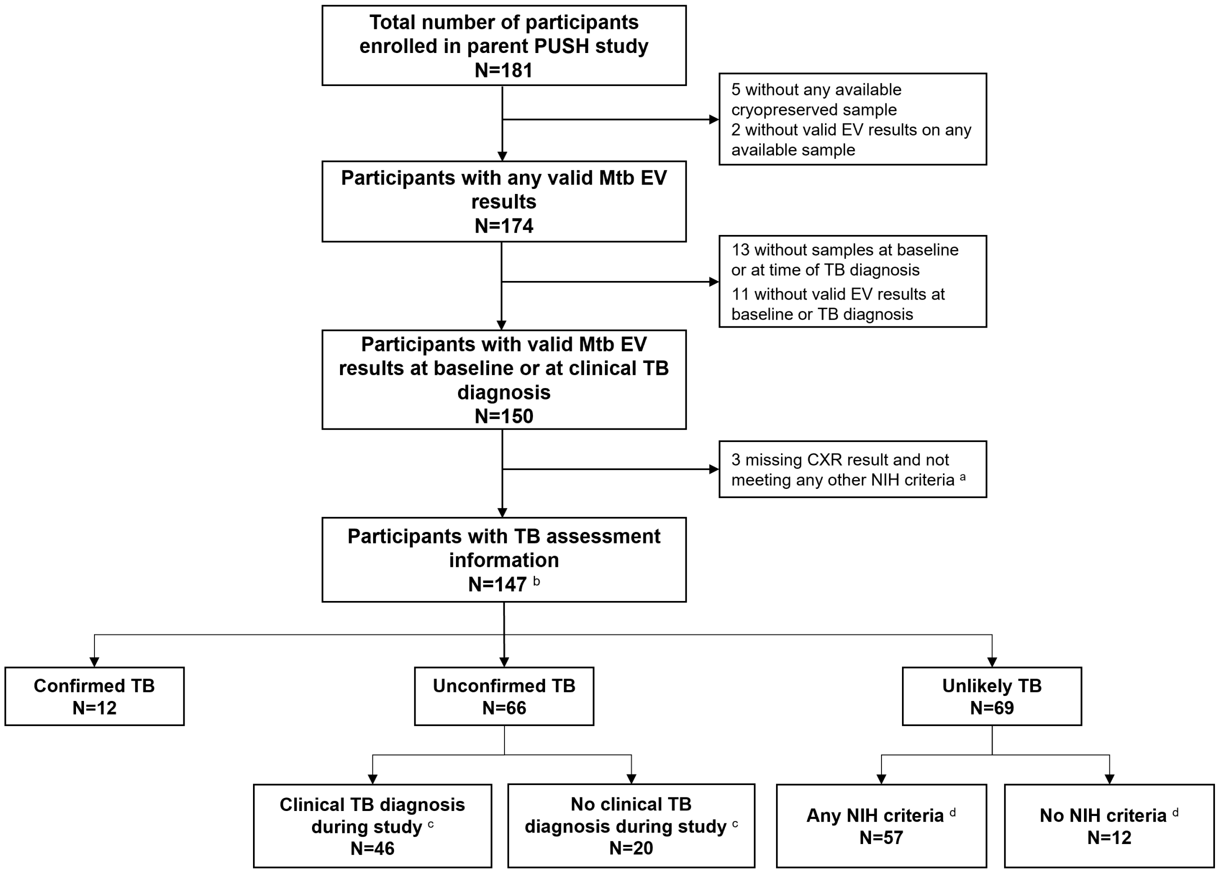

Supplementary Fig. 15 l Study flow of HIV+ hospitalized ART-naïve children evaluated for TB and *Mtb* EV. TB classifications made post-hoc and based on NIH classifications: Confirmed TB: Bacterial confirmation obtained with positive respiratory sample Xpert or culture result. Unconfirmed TB: Bacterial confirmation NOT obtained AND at least 2: suggestive symptoms of TB, CXR consistent with TB, close TB exposure or immunologic evidence of M. tuberculosis infection, TB treatment response. Unlikely TB: Not fitting in any of the TB diagnosis criteria. (Graham et al. CID 2015).

[a] 3 participants with Unlikely TB but missing CXR, therefore unable to further categorize.
[b] Includes 137 with samples analyzed at baseline; 7 at later visit at time of TB diagnosis and 3 with unlikely TB with first samples available <14 days after enrollment
[c] Assessed during study: Clinical TB diagnosis – initiated TB treatment; No clinical TB diagnosis – did not initiate TB treatment
[d] NIH criteria: TB symptoms (persistent cough (>14 days), fever (>7 days), failure to thrive, lethargy (>7 days)), CXR consistent with TB, or TB exposure within last 2 years or TST ≥5mm. Failure to thrive=wasted (WHZ<-2 or MUAC<12.5) or underweight (WAZ<-2) at enrollment.

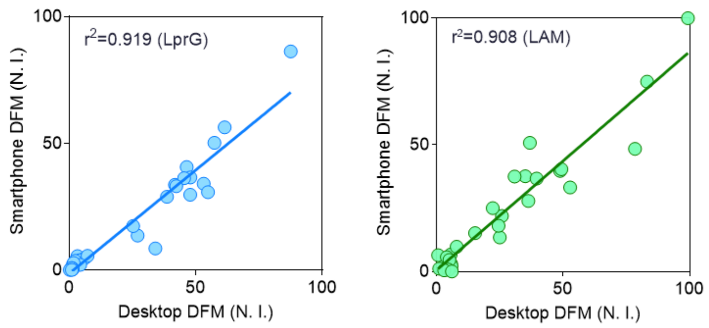

Supplementary Fig. 16 l Comparison of EV LAM and EV LprG NEI signal obtained using images captured by a desktop DFM and a portable smartphone-based DFM device from EVs of 15 TB and 15 non-TB children. Linear regression lines and squared Pearson correlation coefficients are shown for each marker.

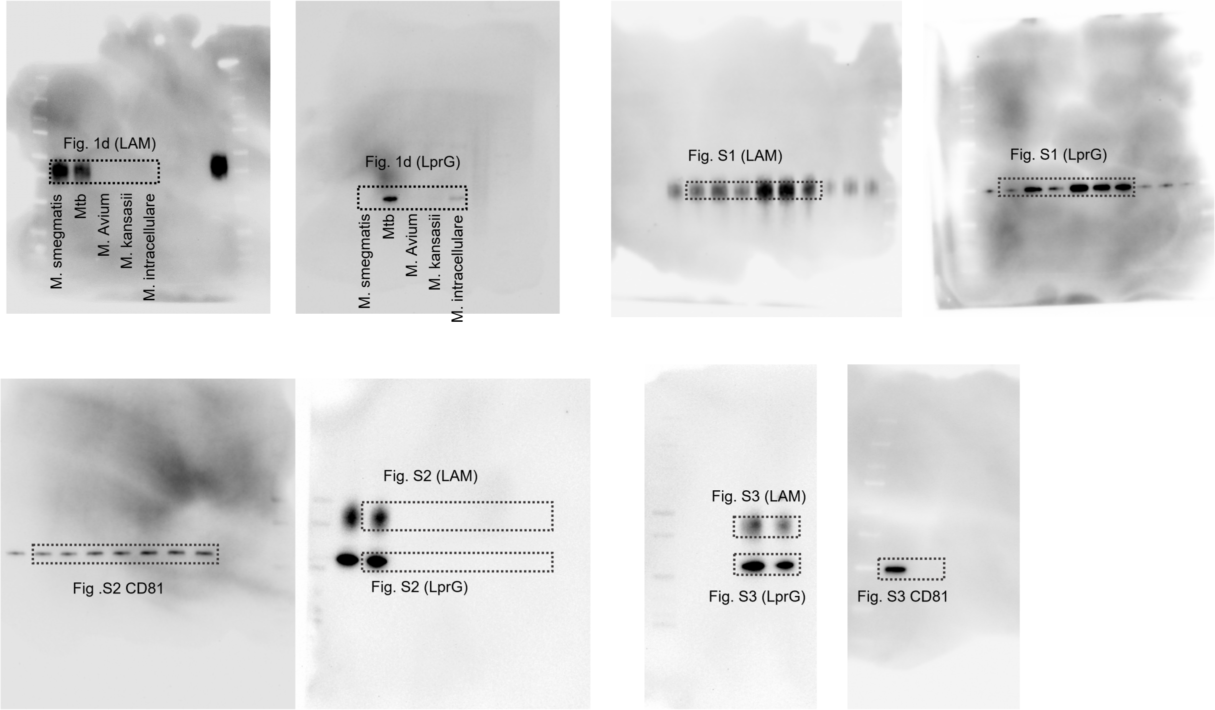

Supplementary Fig. 17 l Original western blot images for Fig. 1d, and for Supplementary Figs. 1–3. The dashed boxes indicate cropping boundaries.

Supplementary Table 1 l Demographics and test data for naïve and *Mtb* aerosol-exposed Rhesus macaques. PTB: pulmonary TB; LTBI: latent TB infection; NA: not available; ND: not detectable, TST, tuberculin skin test; CXR, chest X-ray; TST and CXR score are defined as previous studies.[2]

NHP ID	Age (years)	Sex	Disease Stage	CRP	CXR	Respiratory samples			TST Status	
				mg/dL	Score	Lung tissue CFU/g (Log10)	Histology (% involvement)	BAL CFU/mL (Log10)	Pre-infection	1 month post-infection
KP77	4.95	M	PTB	31.2	1	5.01	58.14	4.04	–	+
KM67	5.05	M	PTB	14.8	3	4.43	51.74	3.34	–	+
KR71	4.98	M	PTB	13.1	2	3.69	22.81	2.85	–	+
KN08	5.1	M	PTB	4.2	2	2.85	34.41	2.18	–	+
KP87	5.04	M	PTB	11.8	2	2.87	22.39	2.60	–	+
JF47	7.32	M	LTBI	<0.5	0	ND	0.2	ND	–	+
HV02	9.34	M	LTBI	<0.5	0	1.78	3.4	ND	–	+
GP50	11.18	M	LTBI	<0.5	0	ND	0.3	ND	–	+
JD72	7.38	M	LTBI	<0.5	0	ND	0.2	ND	–	+
NA01	17.16	F	*Mtb* Naïve	<0.5	0	NA	NA	0	–	–
FP15	12.53	M	*Mtb* Naïve	<0.5	0	NA	NA	0	–	–
LD09	3.41	M	*Mtb* Naïve	<0.5	0	NA	NA	0	–	–
LE99	3.27	M	*Mtb* Naïve	<0.5	0	NA	NA	0	–	–

Supplementary Table 2 | Characteristics of children NHL training cohort (N=20). The numbers are provided as medians, with interquartile ranges or the percentage of participants with positive results.

**Characteristics**	**Number of children**	**Total (20 Children)**	**Confirmed TB (15 Children)**	**Non-TB (5 Children)**
Age (years)	20	11.5 (7.3, 14.5)	13.2 (8.1, 15.4)	7.0 (2.0, 9.9)
Female sex	20	8 (40)	7 (46.7)	1 (20.0)
**Number of Children**				
With TB contact	20	2 (10.0)	2 (13.3)	0 (0.0)
With immunologic evidence of TB	20	14 (70.0)	14 (93.3)	0 (0.0)
With positive respiratory *Mtb* culture or Xpert	20	15 (75.0)	15 (100.0)	0 (0.0)

Supplementary Table 3 | Clinical information from the NHL training cohort of pediatric TB cases (N=15) and controls (N = 5). PTB: pulmonary TB, EPTB: extrapulmonary TB; NA: not applicable; TBTx: TB treatment; 2RHZE: 2-month intensive treatment with rifampin (R), isoniazid (H), pyrazinamide (Z), and ethambutol (E) with the addition of streptomycin (S) for cases treated with 2RHZES; 4RH/4RHE or 10 RH: 4-month or 10-month continuation phase treatment with rifampin (R), isoniazid (H) with the addition of ethambutol (E) for indicated cases treated with 4RHE.

ID	Study group	Age (yrs)	Sex	TB contact	History of TB disease	Diagnosis	TBTx	TBTx regimen	Xpert or Xpert Ultra	Culture	IGRA
1	Control	11.6	M	No	No	Broncho-pulmonary infection	No	NA	–	–	–
2	Control	7.0	F	No	No	Broncho-pulmonary infection	No	NA	–	–	–
3	Control	2.0	M	No	No	Broncho-pulmonary infection	No	NA	–	–	–
4	Control	1.6	M	No	No	Broncho-pulmonary infection	No	NA	–	–	–
5	Control	9.94	M	No	No	Broncho-pulmonary infection	No	NA	–	–	–
6	TB	6 mo	F	No	No	PTB and EPTB	Yes	2RHZE/4RH	–	+	+
7	TB	14.9	F	No	No	EPTB	Yes	2RHZE/4RH	–	+	+
8	TB	7.5	F	Yes	No	PTB and EPTB	Yes	2RHZE/10RH	+	+	+
9	TB	8.1	M	No	No	PTB	Yes	2RHZE/4RH	-	+	+
10	TB	14.0	M	No	No	PTB	Yes	2RHZES/4RH	+	+	+
11	TB	9.8	M	No	No	EPTB	Yes	2RHZE/4RH	-	+	+
12	TB	15.4	F	No	No	PTB	Yes	2RHZE/4RHE	+	+	–
13	TB	13.1	M	No	No	PTB	Yes	2RHZE/4RH	+	+	+
14	TB	17.1	M	Yes	No	PTB and EPTB	Yes	2RHZE/4RHE	+	+	+
15	TB	14.1	M	No	No	PTB	Yes	2RHZE/4RHE	+	+	+
16	TB	15.8	F	No	No	PTB	Yes	2RHZE/4RH	+	+	+
17	TB	13.2	F	No	No	PTB and EPTB	Yes	2RHZE/4RHE	+	+	+
18	TB	11.3	M	No	No	PTB and EPTB	Yes	2RHZE/4RH	–	+	+
19	TB	15.6	F	No	No	PTB	Yes	2RHZE/4RH	–	+	+
20	TB	2.3	M	No	No	PTB and EPTB	Yes	2SRHZE/10RH	+	+	+

Supplementary Table 4 I Baseline characteristics of Children from DR cohort (N=43). The numbers are provided as medians, with interquartile ranges or the percentage of participants with positive results. TST, tuberculin skin test; IGRA, Interferon-Gamma Release Assay. CXR, chest X-ray.

**Characteristics**	**Number of children with results**	**Total (43 Children)**	**Confirmed TB (17 Children)**	**Unconfirmed TB (11 Children)**	**Non-TB Control (15 Children)**
Age (years)	43	9 (3.5, 13.5)	11 (4, 14)	9 (5, 14.5)	7 (3.5, 10)
Female sex	43	27 (59.1)	14 (82.4)	6 (54.6)	7 (46.7)
**Number of Children**					
With signs/symptoms suggestive of TB	43	26 (60.5)	15 (88.2)	9 (81.8)	2 (13.3)
With positive TST or IGRA	40	16 (40.0)	12 (70.6)	4 (36.4)	0 (0.0)
With TB exposure	3	3 (7.0)	1 (5.9)	1 (9.1)	1 (6.7)
With CXR suggestive of TB	42	24 (55.8)	14 (82.4)	10 (90.9)	0 (0.0)
With positive respiratory *Mtb* culture	11	7 (16.3)	7 (41.2)	0 (0.0)	0 (0.0)
With positive Xpert	25	16 (37.2)	16 (94.1)	0 (0.0)	0 (0.0)

Supplementary Table 5 I Diagnostic performance of *Mtb* EV for TB among of children from DR cohort n=43[a]. Number and percentage of children positive or negative according to *Mtb* EV, 95% confidence intervals for the sensitivities and specificities, and interquartile ranges for the *Mtb* EV levels are provided.

	**Confirmed TB (17 Children)**	**Unconfirmed TB (11 Children)**	**Non-TB control[c] (15 Children)**
Number of children positive according to *Mtb* EV, %	15 (88.2)	9 (81.8)	2 (13.3)
Number of children negative according to *Mtb* EV, %	2 (11.8)	2 (18.2)	13 (86.7)
Sensitivity, %	88.2 (63.6, 98.5)	81.8 (48.2, 97.7)	—
Specificity, %	—		86.7 (59.5, 98.3)
Mtb EV levels median	16.1 (9.3, 21.1)	13.3 (5.7, 25.5)	2.5 (0.5, 3.5)
P-value[b]	p<0.001	p=0.0028	Reference

[a] Assessed based on international consensus clinical case definitions for pediatric TB[3]
[b] Two-sided Wilcoxon rank sum test compared to Non-TB control.
[c] Including three children with one of the criteria used for TB diagnosis.

Supplementary Table 6 | Criteria for pediatric TB case definitions[3].

Confirmed TB
Bacteriologic confirmation (culture or Xpert *MTB*/RIF) from respiratory specimen
**Unconfirmed TB**

Bacteriologic confirmation NOT obtained and at least 2 of the following:

- Symptoms suggestive of TB
  - Persistent cough (cough>2 weeks, non-remitting cough)
  - Weight loss/Failure to thrive (unexplained weight loss, >5% reduction in weight in last 3 months, deviation from growth trajectory, crossing of percentile lines in prior 3 months, WAZ <=-2 or WHZ <=-2 in absence of prior growth trajectory AND not responding to nutritional rehabilitation or ART if HIV+)
  - Persistent unexplained fever (fever >1 week reported by guardian, or objectively recorded at least once (>38.0)
  - Persistent unexplained lethargy or decreased playfulness reported by caregiver
  - Infants 0-16 days: neonatal pneumonia, unexplained hepatosplenomegaly or sepsis
- CXR consistent with TB
  - Read by 2 independent, blinded reviewers; Quality of CXR indicated; Standardized forms with predetermined terminology and yes/no options for CXR reader.
  - Example criteria from SATVI:
    - 1) Airway compression or tracheal displacement
    - 2) Soft tissue density suggestive of lymphadenopathy
    - 3) Air space opacification
    - 4) Nodular picture (miliary or larger, widespread and bilateral)
    - 5) Pleural effusion
    - 6) Cavities
    - 7) Calcified parenchyma (Ghon focus)
    - 8) Vertebral spondylitis
- Close TB exposure or evidence of *Mtb* infection (TST or IGRA)
- Positive response to TB treatment (clinical features suggestive of TB at baseline have improved and no new clinical feature suggestive of TB)
- Expert panel findings for a TB related TB-death

Unlikely TB
Bacteriologic confirmation NOT obtained, criteria for unconfirmed TB NOT met

*Children were classified as having confirmed TB, unconfirmed TB, or unlikely TB, based the 2015 NIH consensus clinical case definitions,[3] which were modified to include a judgement of probable/possible TB-related death (Supplementary Table 7) as an alternate factor for unconfirmed TB classification.

Supplementary Table 7 | Criteria to Assess Relatedness of Death to TB by Expert Review Panel.

Classification	Definition
Unlikely TB death	Child does not meet criteria for unconfirmed TB prior to death and has an alternate etiology of death (presumed or confirmed). For cases in which the alternate etiology is presumed but the expert panel is unable to definitively rule out intrathoracic TB (respiratory symptoms and/or diagnosis of pneumonia), the death is determined to be unlikely related to TB if the alternate etiology is considered more likely than TB.
Possible TB death	Child meets classification criteria for unconfirmed TB prior to death.<br>If the child does not meet unconfirmed TB classification criteria, their death is considered as possibly related to TB if their clinical presentation is compatible with intrathoracic TB (respiratory symptoms and/or diagnosis of pneumonia) and no alternate diagnosis is more likely.
Likely TB death	Child meets classification criteria for confirmed TB.

Supplementary Table 8 | Criteria employed for reclassification from unlikely TB to unconfirmed TB. Criteria for judgement of possible TB death are listed in Supplementary Table 7. Reclassification occurred post-hoc after-parent study completion.

Study ID	Initial criterion	Subsequent criteria	*Mtb* EV+
1	TB Symptoms	TB Symptoms<br>Possible TB death	Y
2	TB Symptoms	TB Symptoms<br>Possible TB death	Y
3	TB Symptoms	TB Symptoms<br>Possible TB death	N
4	TB Contact	TB Contact<br>Possible TB death	Y
5	TB Symptoms	TB Symptoms<br>TBTx response	Y
6	TB Symptoms	TB Symptoms<br>TBTx response	Y
7	CXR findings	CXR findings<br>TBTx response	Y
8	CXR findings	CXR findings<br>TBTx response	Y
9	None	TB Symptoms<br>TBTx response	N
10	None	TB Symptoms<br>CXR findings	Y

Supplementary Table 9 I Estimated component cost for the portable DFM device.

**Components**	**Price (US dollars)**
Mechanical parts of translation stage	60.00
3D printed parts	10.00
Objective lens	20.00
Darkfield condenser	40.00
Motor × 2	20.00 × 2
Motor driver × 2	14.95 × 2
IOIO-OTG electronic board	40.95
Bluetooth Dongle	7.96
Rechargeable Battery sets	46.00
Switch	1.00
Snap action microswitch	5.90
Charging port	2.00
Total	**263.71**

References

1. Prados-Rosales, R., et al. Mycobacteria release active membrane vesicles that modulate immune responses in a TLR2-dependent manner in mice. *J. Clin. Investig.* **121**, 1471-1483 (2011).
2. Foreman, T.W.*, et al.* CD4+ T-cell–independent mechanisms suppress reactivation of latent tuberculosis in a macaque model of HIV coinfection. *Proc. Natl. Acad. Sci. U.S.A* **113**, E5636-E5644 (2016).
3. Graham, S.M.*, et al.* Clinical case definitions for classification of intrathoracic tuberculosis in children: an update. *Clin. Infect. Dis.* **61**, S179-S187 (2015).
