# Supplementary material for: Diagnosis of paediatric tuberculosis by optically detecting two virulence factors on extracellular vesicles in blood samples
Source: Nat Biomed Eng. 2022 Aug 19;6(8):979–91. doi: 10.1038/s41551-022-00922-1 (PMC9391224; doi:10.1038/s41551-022-00922-1)
Supplement: Supplementary file 2 — Reporting Summary [file 41551_2022_922_MOESM2_ESM.pdf]

nature research

Corresponding author(s): Tony Hu

Last updated by author(s): Mar 12, 2022

Reporting Summary

Nature Research wishes to improve the reproducibility of the work that we publish. This form provides structure for consistency and transparency in reporting. For further information on Nature Research policies, see our Editorial Policies and the Editorial Policy Checklist.

Statistics

For all statistical analyses, confirm that the following items are present in the figure legend, table legend, main text, or Methods section.

n/a	Confirmed	
☐	☒	The exact sample size (*n*) for each experimental group/condition, given as a discrete number and unit of measurement
☐	☒	A statement on whether measurements were taken from distinct samples or whether the same sample was measured repeatedly
☐	☒	The statistical test(s) used AND whether they are one- or two-sided<br>*Only common tests should be described solely by name; describe more complex techniques in the Methods section.*
☐	☒	A description of all covariates tested
☐	☒	A description of any assumptions or corrections, such as tests of normality and adjustment for multiple comparisons
☐	☒	A full description of the statistical parameters including central tendency (e.g. means) or other basic estimates (e.g. regression coefficient) AND variation (e.g. standard deviation) or associated estimates of uncertainty (e.g. confidence intervals)
☐	☒	For null hypothesis testing, the test statistic (e.g. *F*, *t*, *r*) with confidence intervals, effect sizes, degrees of freedom and *P* value noted<br>*Give P values as exact values whenever suitable.*
☒	☐	For Bayesian analysis, information on the choice of priors and Markov chain Monte Carlo settings
☒	☐	For hierarchical and complex designs, identification of the appropriate level for tests and full reporting of outcomes
☐	☒	Estimates of effect sizes (e.g. Cohen's *d*, Pearson's *r*), indicating how they were calculated

Our web collection on statistics for biologists contains articles on many of the points above.

Software and code

Policy information about availability of computer code

Data collection	NIS-Elements AR 5.0
Data analysis	GraphPad Prism 9.0, Image J 1.53, Microsoft Office 365 Excel, Matlab R2020a, SAS OnDemand version 9.4, Stata 17.0. Source code used in this work is available for non-commercial purposes from the corresponding author on request.

For manuscripts utilizing custom algorithms or software that are central to the research but not yet described in published literature, software must be made available to editors and reviewers. We strongly encourage code deposition in a community repository (e.g. GitHub). See the Nature Research guidelines for submitting code & software for further information.

Data

Policy information about availability of data

All manuscripts must include a data availability statement. This statement should provide the following information, where applicable:

- Accession codes, unique identifiers, or web links for publicly available datasets
- A list of figures that have associated raw data
- A description of any restrictions on data availability

The data supporting the results in this study are available within the paper and its Supplementary Information. Source data is provided with this paper. The raw and analysed datasets generated during the study are too large to be publicly shared, yet they are available for research purposes from the corresponding author on reasonable request. De-identified patient data are available from the corresponding author, subject to IRB approval.

Field-specific reporting

Please select the one below that is the best fit for your research. If you are not sure, read the appropriate sections before making your selection.

☒ Life sciences ☐ Behavioural & social sciences ☐ Ecological, evolutionary & environmental sciences

For a reference copy of the document with all sections, see nature.com/documents/nr-reporting-summary-flat.pdf

Life sciences study design

All studies must disclose on these points even when the disclosure is negative.

Sample size	No statistical methods were used to predetermine sample sizes, which for each retrospective cohort were determined by the number of patients with sample available.
Data exclusions	No data ware excluded from the analyses.
Replication	Information on experimental replication is provided in Methods and in figure legends. Replicate numbers were chosen on the basis of the minimum number required to detect differences between groups in cell-culture experiments, owing to constraints involved with the handling of increasing numbers of cells infected with Mycobacterium tuberculosis. Studies performed with archived samples from retrospective non-human-primate and patient cohorts employed all available samples, which were analysed using triplicate technical replicates.
Randomization	The cell samples and animals used in this study were randomly allocated to each experimental group prior to treatment. All individuals in the analysed study cohorts were categorized using predefined classification criteria, and all available samples from these individuals were analysed as described.
Blinding	The investigators were not blinded for the samples analysed in the training cohort, cell-culture studies and NHP cohort, but were blinded to the identification of the DR and PUSH cohort samples.

Reporting for specific materials, systems and methods

We require information from authors about some types of materials, experimental systems and methods used in many studies. Here, indicate whether each material, system or method listed is relevant to your study. If you are not sure if a list item applies to your research, read the appropriate section before selecting a response.

Materials & experimental systems

n/a	Involved in the study	
☐	☒	Antibodies
☐	☒	Eukaryotic cell lines
☒	☐	Palaeontology and archaeology
☐	☒	Animals and other organisms
☐	☒	Human research participants
☐	☒	Clinical data
☒	☐	Dual use research of concern

Methods

n/a	Involved in the study	
☒	☐	ChIP-seq
☒	☐	Flow cytometry
☒	☐	MRI-based neuroimaging

Antibodies

Antibodies used	Mouse antibodies specific for human CD81 (Clone 5A6, Cat#: 349502) were purchased from BioLegend, mouse antibodies for human CD9 (Clone ALB 6, Cat#:sc-59140) and CD63 (Clone MX-49.129.5, Cat#:sc-5275) are from Santa Cruz Biotechnology, Inc., biotinylated mouse anti-human CD81 (Clone 5A6, Cat#: 349502), anti-CD9 (Clone HI9a , Cat#: 312102) and anti-CD63 (Clone H5C6, Cat#:353018) were purchased from BioLegend, USA.<br>Anti-LAM antibodies (Clone CS35, Cat#:NR-13811 and Clone CS40, Cat#:NR-13812) and anti-LprG antibodies (Clone A Cat#:NR-13806 and Clone B, Cat#:NR-51133) were obtained from BEI Resources. Anti-LAM antibody (A194-01, CAT#:HPAB-M0560-YC) was purchased from Creative Biolabs, USA.<br>Specific antibodies for Mtb LpqH (Clone IT54, Cat#:NR-13792), MPT64 (Clone B, Cat#:NR-50704), MPT51 (Clone B, Cat#:NR-50106), MPT32 (Rv1860, Cat#: NR-13817), Ag85c (Clone , Cat#:NR-13800), Ag85b (Clone , Cat#: NR-19362) and KatG (Clone B, Cat#: NR-50101) were obtained from BEI Resources.<br>HRP-labeled goat anti-mouse (Cat#: 115-035-166)/human (Cat#:109-035-170)/rabbit (111-035-144) IgG are from Jackson ImmunoResearch Laboratories, USA.
Validation	All antibodies were obtained from commercial manufacturers, and employed without further validation. Validation statements are provided on the manufacturer's websites.

Eukaryotic cell lines

Policy information about cell lines

Cell line source(s)	THP-1 monocytes were purchased from the American Type Culture Collection (ATCC; Manassas, VA).
Authentication	Cell lines were obtained from the ATCC and used without further authentication.
Mycoplasma contamination	All cell lines tested negative for mycoplasma contamination.
Commonly misidentified lines (See ICLAC register)	No misidentified cell lines were used.

Animals and other organisms

Policy information about studies involving animals; ARRIVE guidelines recommended for reporting animal research

Laboratory animals	Specific-pathogen-free, retrovirus-free, and mycobacteria-naive adult rhesus macaques were assigned to three experimental groups that received different Mtb exposures.
Wild animals	The study did not involve wild animals.
Field-collected samples	The study did not involve samples collected from the field.
Ethics oversight	No live rhesus macaques were used in this study. The cryopreserved NHP plasma was archived material obtained from NHPs infected with Mtb in previously reported studies (approved by the respective Institutional Animal Care and Use Committee as well as by the Institutional Biosafety Committee at Tulane National Primate Research Center and Southwest National Primate Research Center).

Note that full information on the approval of the study protocol must also be provided in the manuscript.

Human research participants

Policy information about studies involving human research participants

Population characteristics	This study analysed archived samples and data collected in three independent studies.<br><br>The Pediatric Urgent vs. post-Stabilization Highly active anti-retroviral therapy initiation (PUSH) study was a randomized controlled trial (NCT02063880) performed in Kenya that evaluated whether urgent (<48 hours) vs. post-stabilization anti-retroviral therapy (7–14 days) improved survival in hospitalized HIV-infected children aged <12 years.<br><br>National Lung Hospital (NLH) cohort: Specimens and associated clinical data were collected 20 children aged ≤17 who consecutively visited the NLH in Ha Noi, Vietnam for clinical assessment and medical evaluation, using a protocol approved by the NLH IRB, which included an amendment approving the analyses performed in this study.<br><br>Dominican Republic (DR) cohort: Specimens and associated clinical data were collected from 48 children aged ≤ 18 years, who were enrolled and consecutively followed at the Robert Reid Children's Hospital or the Hugo Mendoza Children's Hospital.
Recruitment	Recruitment details for these independent studies are briefly described in Methods, and fully described in publications describing these studies (cited in Methods).
Ethics oversight	Permission to analyse the NLH cohort samples and clinical data at Tulane University was granted by the NLH Institutional Review Board.<br>Permission to analyse the PUSH cohort samples and clinical data at Tulane was granted by the IRB of Kenyatta National Hospital and the University of Nairobi.<br>Permission to analyse the DR cohort samples and clinical data at Tulane was granted by the IRB of the University Dominicana O&M.

Note that full information on the approval of the study protocol must also be provided in the manuscript.

Clinical data

Policy information about clinical studies

All manuscripts should comply with the ICMJE guidelines for publication of clinical research and a completed CONSORT checklist must be included with all submissions.

Clinical trial registration	This study did not involve a clinical trial. It used archived samples and clinical data from a completed clinical trial (PUSH).
Study protocol	—
Data collection	—
Outcomes	—
